# Systematic identification of terpene synthases from sacred lotus (*Nelumbo nucifera*) and heterologous biosynthesis of the insecticidal and antimicrobial compound γ-eudesmol

**DOI:** 10.1093/hr/uhaf191

**Published:** 2025-07-28

**Authors:** Zhenni Xu, Xueting Fang, Yao Zhi, Xiaochun Xiao, Jing Yang, Jie Hu, Hangzhi Zhu, Fangfang Chen, Weijia Cheng, Tiangang Liu, Li Lu

**Affiliations:** Department of Urology, Zhongnan Hospital of Wuhan University, Hubei Provincial Research Center for Basic Biological Science, School of Pharmaceutical Sciences, Wuhan University, Wuhan 430071, China; State Key Laboratory of Hybrid Rice, Hubei Hongshan Laboratory, School of Pharmaceutical Sciences, Wuhan University, Wuhan 430071, China; Department of Urology, Zhongnan Hospital of Wuhan University, Hubei Provincial Research Center for Basic Biological Science, School of Pharmaceutical Sciences, Wuhan University, Wuhan 430071, China; State Key Laboratory of Hybrid Rice, Hubei Hongshan Laboratory, School of Pharmaceutical Sciences, Wuhan University, Wuhan 430071, China; Department of Urology, Zhongnan Hospital of Wuhan University, Hubei Provincial Research Center for Basic Biological Science, School of Pharmaceutical Sciences, Wuhan University, Wuhan 430071, China; Wuhan Hesheng Technology Co., Ltd., Wuhan 430074, China; Department of Urology, Zhongnan Hospital of Wuhan University, Hubei Provincial Research Center for Basic Biological Science, School of Pharmaceutical Sciences, Wuhan University, Wuhan 430071, China; State Key Laboratory of Hybrid Rice, Hubei Hongshan Laboratory, School of Pharmaceutical Sciences, Wuhan University, Wuhan 430071, China; School of Life Sciences and Biotechnology, Shanghai Jiao Tong University, Shanghai 200030, China; Department of Urology, Zhongnan Hospital of Wuhan University, Hubei Provincial Research Center for Basic Biological Science, School of Pharmaceutical Sciences, Wuhan University, Wuhan 430071, China; State Key Laboratory of Hybrid Rice, Hubei Hongshan Laboratory, School of Pharmaceutical Sciences, Wuhan University, Wuhan 430071, China; Department of Urology, Zhongnan Hospital of Wuhan University, Hubei Provincial Research Center for Basic Biological Science, School of Pharmaceutical Sciences, Wuhan University, Wuhan 430071, China; State Key Laboratory of Hybrid Rice, Hubei Hongshan Laboratory, School of Pharmaceutical Sciences, Wuhan University, Wuhan 430071, China; Department of Urology, Zhongnan Hospital of Wuhan University, Hubei Provincial Research Center for Basic Biological Science, School of Pharmaceutical Sciences, Wuhan University, Wuhan 430071, China; State Key Laboratory of Hybrid Rice, Hubei Hongshan Laboratory, School of Pharmaceutical Sciences, Wuhan University, Wuhan 430071, China; Department of Urology, Zhongnan Hospital of Wuhan University, Hubei Provincial Research Center for Basic Biological Science, School of Pharmaceutical Sciences, Wuhan University, Wuhan 430071, China; State Key Laboratory of Hybrid Rice, Hubei Hongshan Laboratory, School of Pharmaceutical Sciences, Wuhan University, Wuhan 430071, China; Department of Urology, Zhongnan Hospital of Wuhan University, Hubei Provincial Research Center for Basic Biological Science, School of Pharmaceutical Sciences, Wuhan University, Wuhan 430071, China; Department of Pharmacy, Renmin Hospital of Wuhan University, Wuhan 430060, China; Wuhan Hesheng Technology Co., Ltd., Wuhan 430074, China; School of Life Sciences and Biotechnology, Shanghai Jiao Tong University, Shanghai 200030, China; Department of Urology, Zhongnan Hospital of Wuhan University, Hubei Provincial Research Center for Basic Biological Science, School of Pharmaceutical Sciences, Wuhan University, Wuhan 430071, China; State Key Laboratory of Hybrid Rice, Hubei Hongshan Laboratory, School of Pharmaceutical Sciences, Wuhan University, Wuhan 430071, China

## Abstract

Sacred lotus is widely used in the agricultural, nutraceutical, and pharmaceutical industries. Terpenes are not only crucial components of sacred lotus essential oil, but also serve as signaling molecules involved in plant–environment interactions. However, the biosynthesis of terpenes in sacred lotus has not yet been reported. Thus, gene-directed heterologous mining and combinatorial biosynthesis methods were used in this study to systematically characterize the function of terpene synthase genes in the sacred lotus. As a result, two monoterpene, 11 sesquiterpene, and three diterpene products were synthesized, and a highly efficient γ-eudesmol synthase was discovered. In addition, a mechanistic study revealed that N314 is the key amino acid responsible for the secondary cyclization that produces γ-eudesmol. *In vitro* assays demonstrated that γ-eudesmol exhibited substantial insecticidal and antimicrobial activities. Furthermore, *de novo* biosynthesis of γ-eudesmol was achieved in a yeast chassis through a series of metabolic engineering strategies, reaching a titer of 801.66 mg/L in a shake flask, the highest yield reported to date. The present study uncovered the biosynthesis of terpenes in sacred lotus, as well as successfully synthesized the bioactive compound γ-eudesmol by synthetic biology. This comprehensive strategy can be readily adapted for investigation and the production of other valuable plant-derived natural products.

## Introduction

Plant-derived natural products play a critical role in pharmaceuticals and healthcare applications within daily life [1]. However, the low abundance of plant secondary metabolites in nature limits their utilization. Fortunately, a synthetic biology-based strategy offers an innovative solution for the development and application of plant-derived natural products [2]. Concisely, heterologous expression of plant biosynthetic genes in microbial chassis allows large-scale compound production *via* fermentation for functional exploration and industrial applications [3]. This strategy circumvents the reliance on plant material and facilitates scalable synthesis of trace bioactive constituents. In recent years, several critical plant-derived pharmaceutical ingredients, such as artemisinin and lycopene, have been industrially produced *via* biosynthetic strategies [4, 5].

The sacred lotus (*Nelumbo nucifera*), a perennial aquatic plant, is considered one of the early-diverging lineages within angiosperms [6]. Additionally, the sacred lotus has a long history of both medicinal and culinary use. Its seeds and rhizomes are nutrient-dense and possess unique health-promoting properties [7], while lotus flower essential oil demonstrates antioxidant and anti-inflammatory bioactivities [8, 9]. Phytochemical analyses reveal that this essential oil contains diverse terpenoids, including linalool, geraniol, perilla alcohol, eudesmol, and kaurenoic acid [10, 11], which collectively contribute to its distinctive fragrance and exhibit potential antimicrobial and anticancer properties [12, 13]. However, the application of lotus-derived terpenoids remains underexplored, and their biosynthetic pathways in *N. nucifera* have yet elucidated.

Terpenoid biosynthesis in plants primarily relies on the mevalonate (MVA) and methylerythritol-4-phosphate pathways to generate the universal precursors dimethylallyl diphosphate (DMAPP) and isopentenyl diphosphate (IPP) [14]. These precursors are subsequently elongated by prenyltransferase (PT), such as geranyl pyrophosphate synthase (GPPS), farnesyl pyrophosphate synthase (FPPS), and geranylgeranyl pyrophosphate synthase (GGPPS), to form geranyl pyrophosphate (GPP), farnesyl pyrophosphate (FPP), and geranylgeranyl pyrophosphate (GGPP), which serve as precursors for monoterpenes, sesquiterpenes, and diterpenes, respectively. Notably, recent studies have reported that plant terpene synthases (TPSs) can also utilize alternative precursors, such as nerolidyl diphosphate (NPP) and *Z,Z*-farnesyl diphosphate (*Z,Z*-FPP), to generate diverse terpenoid scaffolds [15–17].

*Saccharomyces cerevisiae* is one of the most widely used eukaryotic expression systems, offering distinct advantages including high biosafety, industrial scalability, and well-characterized genetics, making it a preferred chassis for food biotechnology and biopharmaceutical production [18]. Through rational metabolic engineering strategies, high-yield production of various terpenoids was achieved in yeast chassis, including patchoulol and valencene [19, 20].

γ-Eudesmol, the most abundant volatile terpenoid component in sacred lotus, is a eudesmane-type sesquiterpenoid [11]. Eudesmane-type sesquiterpenoids are widely distributed in nature and belong to a class of bicyclic sesquiterpenes, which often exhibit diverse bioactivities. γ-Eudesmol has been identified as a major volatile constituent in the essential oils of dozens of plant species across multiple families, including *Eugenia stipitata*, *Tanacetum dolichophyllum* and *Scutellaria brevibracteata* [21–23]. Pharmacological studies have shown that γ-eudesmol demonstrates growth inhibitory activity against cancer cell lines, such as HepG2 and B16-F10 [24]. However, the plant-related bioactivities and ecological functions of γ-eudesmol as a monomer remain understudied, probably due to the high cost and challenges associated with isolating γ-eudesmol from plant essential oils.

In this study, we systematically characterized terpene synthase genes in *N. nucifera* and identified a novel γ-eudesmol synthase with exceptional catalytic activity. Through site-directed mutagenesis, we elucidated a key amino acid residue responsible for product cyclization. *In vitro* bioactivity assays demonstrated that γ-eudesmol exhibits antimicrobial and insecticidal properties. Based on these findings, we achieved *de novo* biosynthesis of γ-eudesmol in an engineered *S. cerevisiae* platform through modular metabolic engineering strategies.

## Results

### Terpene synthase gene family in sacred lotus

**Figure 1:**
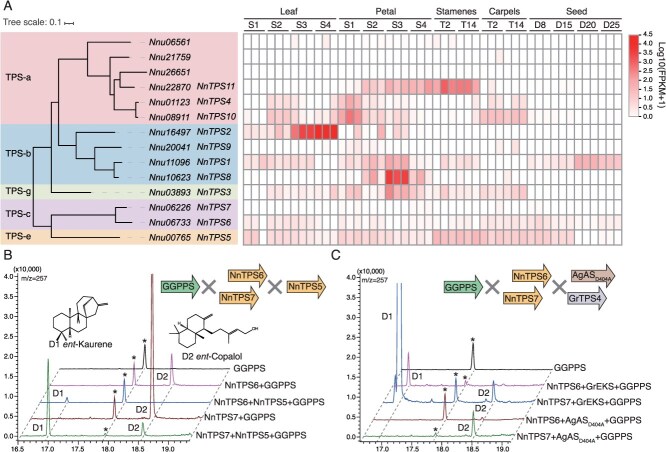
Phylogenetic analysis and functional characterization of labdane-type diterpene synthases in sacred lotus. (A) Phylogenetic tree and heatmap of TPS genes expression in *N. nucifera.* Transcriptome data were derived from NCBI (Table S3). (B) Gas-chromatography (GC) spectrum profile of products from NnTPS6 and NnTPS7 co-expressed with NnTPS5 and GGPPS. (C) GC spectrum profile of products from NnTPS6 and NnTPS7 co-expressed with GrEKS/AgAS_D404A_ and GGPPS. D1, *ent*-kaurene; D2, *ent*-copalol. *Ent*-Kaurene (D1) was identified by comparison to the National Institute of Standards and Technology (NIST17) library; *ent*-Copalol (D2) was identified by comparison to the standards. Asterisks indicate nonspecific products.

Based on the *N. nucifera* genome [25], we identified 20 putative TPS genes by scanning all predicted amino acid sequences against the two conserved domains (PF03936 and PF01397). Therein, five genes were excluded because of their short protein length (<400 amino acids), while two TPSs with 100% identical proteins were merged into one. By constructing a phylogenetic tree, the remaining 14 TPSs could be assigned to five subfamilies: TPS-a (6), TPS-b (4), TPS-g (1), TPS-c (2), and TPS-e (1) (Fig. 1A; Tables S1 and S2). Compared to the TPS genes from *Arabidopsis thaliana* (31) [26], *Oryza sativa* (44) [27], *Vitis vinifera* (44) [28], and *Solanum lycopersicum* (29) [29], the sacred lotus was found to have a smaller number of TPS gene sequences and showed distinct sequences (Fig. S1). By integrating transcriptome data from various tissue parts and different developmental stages of the sacred lotus, we found that *Nnu26651*, *Nnu06561*, and *Nnu21759* were not expressed and were therefore excluded from further study. While *Nnu06733* (TPS-c) and *Nnu00765* (TPS-e) were expressed in almost all samples, the rest TPS genes showed a specific expression pattern in the petals, stamens, or fruit coat (Fig. 1A; Table S3). The varying expression patterns imply that the terpenes they produce may have distinct functions throughout the development of the sacred lotus, including both primary and specialized metabolites.

To fully explore the catalytic potential of these TPS enzymes, we employed a heterologous expression approach, which can overcome the usual limitations of substrate supply and cellular compartmentalization found in the *in vivo* environment. A genetically modified yeast strain, JCR27, engineered to proficiently synthesize terpene precursors IPP and DMAPP by increasing the activity of key enzymes in the MVA pathway [30–32], was utilized as the host for the experiments. The 11 selected TPS genes were manually verified based on transcriptome data and codon optimized for expression in yeast.

### Functional characterization of labdane-type diterpene synthases in sacred lotus

In angiosperms, labdane-type diterpenes are formed by the sequential activity of two monofunctional TPSs, which act as pairs of Class II and Class I enzymes. The Class II and Class I TPSs are categorized under the TPS-c and TPS-e/f clades of the plant TPS superfamily, respectively [33]. NnTPS6 and NnTPS7 belong to the TPS-c subfamily, both containing γβα domains, with the β domain featuring the characteristic motif DxDD of Class II TPS enzymes (Fig. S2B and C). Despite sharing 79% protein similarity, their expression patterns are markedly different. *NnTPS6* is expressed in nearly all tissues, including seeds, reproductive organs, and leaves, whereas *NnTPS7* exhibited consistently low expression levels in all analyzed tissues (Figs 1A and S3). NnTPS5 is a member of the TPS-e subfamily, containing γβα domains with the characteristic DDxxD motif in its α domain (Fig. S2A). As the sole TPS-e gene, *NnTPS5* is expressed in nearly all tested tissues (Figs 1A and S3). When NnTPS6 was co-transformed with GGPPS into the JCR27 yeast strain, an additional peak (**D2**) was observed at a retention time of 18.515 min compared to the control strain transformed with GGPPS alone. The new product was identified as *ent*-copalol (Figs 1B and S4). Furthermore, when combining NnTPS6, NnTPS5, and GGPPS, a new product (**D1**) can be identified as *ent*-kaurene (Figs 1B and S4). NnTPS7 yielded similar results, *ent*-kaurene product can be identified when NnTPS7 was combined with NnTPS5 and GGPPS. However, the production level of *ent*-kaurene was notably higher in NnTPS7, compared to the yield obtained with NnTPS6 (Fig. 1B).

In addition, we co-expressed NnTPS6 and NnTPS7 with substrate-specific Class I terpene synthases to differentiate the chiral configurations of their enzymatic products. GrEKS is a product-specific *ent*-kaurene synthase concurrent with known *ent*-copalyl diphosphate (*ent*-CPP) synthase involved in gibberellin biosynthesis [34]. And a single mutant of abietadiene synthase (AgAS_D404A_) from *Abies grandis* accepting (+)-CPP as substrate [35]. With combinations of NnTPS6 and NnTPS7 with GrEKS, we detected the production of *ent*-kaurene in both cases, despite there were significant differences in yield (Fig. 1C). However, only the production of *ent*-copalol can be detected when they are combined with AgAS_D404A,_ while no derivatives of (+)-CPP were observed (Fig. 1C). Therefore, NnTPS6 and NnTPS7 are both *ent*-CPP synthases, and NnTPS5 (TPS-e) exclusively produces *ent*-kaurene, the precursor of gibberellins.

### Systematic characterization of class I terpene synthases in sacred lotus

**Figure 2:**
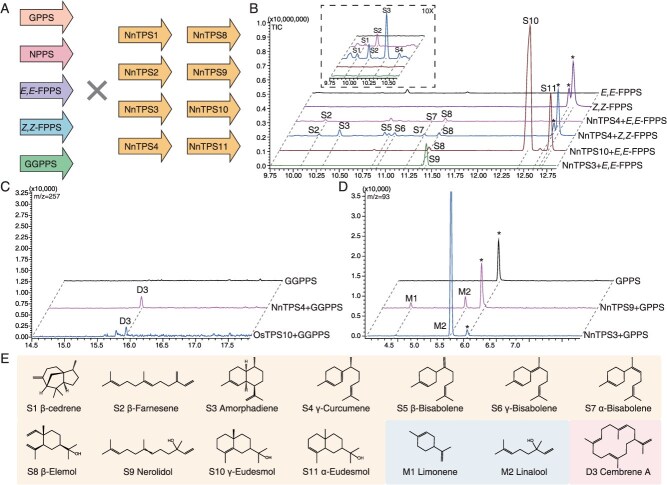
Systematic characterization of Class I terpene synthases in sacred lotus. (A) Schematic diagram of TPS and PT co-expression. (B) GC spectrum profile of products from NnTPS4, NnTPS10 and NnTPS3 co-expressed with *E,E*-FPPS or *Z,Z*-FPPS. (C) GC spectrum profile of products from NnTPS4 and OsTPS10 (GeneID: ABF95916) co-expressed with GGPPS. (D) GC spectrum profile of products of NnTPS9 and NnTPS3 co-expressed with GPPS. (E) Chemical structures of terpenoid products by *N. nucifera* terpene synthases. β-elemol (S8) and γ-eudesmol (S10) were identified by nuclear magnetic resonance; β-cedrene (S1), amorphadiene (S3), α-eudesmol (S11) and *ent-*kaurene (D1) were identified by comparison to the NIST17 library; β-farnesene (S2), γ-curcumene (S4), β-bisabolene (S5), γ-bisabolene (S6), α-bisabolene (S7), nerolidol (S9), limonene (M1), linalool (M2), *ent*-copalol (D2) and cembrene A (D3) were identified by comparison to standards. Asterisk indicates unspecific products.

TPS genes in the TPS-a/b/g clades are classified as Class I TPS and showed diverse functions in the synthesis of mono-, sesqui-, or diterpenes, playing an important role in plant specialized metabolism [33]. To evaluate the ability of these TPS genes in different types of terpene precursor utilization, we co-expressed them with five distinct PT genes, including *GPPS*, *NPPS*, *E,E-FPPS*, *Z,Z-FPPS*, and *GGPPS* (Fig. 2A; Table S4).

As a result, NnTPS4 (TPS-a) exhibited broad substrate specificity (Fig. 2B and C). When co-expressed with *E,E*-FPPS, it produces β-elemol (**S8**), along with the linear sesquiterpene β-farnesene (**S2**) and the 1,6-cyclization product α-bisabolene (**S7**) (Figs 2B and S4). When co-expressed with *Z,Z*-FPPS, it generates amorphadiene (**S3**), a precursor for artemisinin. Interestingly, co-expression of NnTPS4 with GGPPS leads to the 1,14-cyclization, producing the macrocyclic diterpene cembrene A (**D3**), which matches the compound previously reported for the rice terpene synthase OsTPS10 [36] (Figs 2C and S4).

When NnTPS10 (TPS-a) is co-expressed with *E,E*-FPPS, it produces a series of hydroxylated sesquiterpene compounds, including β-elemol (**S8**), γ-eudesmol (**S10**), and α-eudesmol (**S11**) (Figs 2B and S4). Notably, γ-eudesmol is produced in high yields (79.0%). To verify the identity of γ-eudesmol, large-scale assays were conducted to obtain sufficient product for nuclear magnetic resonance (NMR) analysis (Figs S5 and S6). This finding aligns with previous reports identifying γ-eudesmol as a major component in the petals and essential oils of lotus flowers [11]. Thus, NnTPS10 is likely responsible for γ-eudesmol production in sacred lotus.

Four TPS genes belonging to the TPS-b subfamily were tested for functionality in combination with five different PT enzymes; however, only NnTPS9 produced monoterpene products when co-expressed with GPPS (Fig. 2D), and limonene (**M1**) and linalool (**M2**) have been identified as monoterpene products. The limonene is the biosynthetic precursor to perilla alcohol, which constitutes the monoterpene components of sacred lotus essential oil [37]. In addition, NnTPS3 (a TPS-g enzyme) produces linear terpene products. When co-expressed with *E,E*-FPPS, it generates the sesquiterpene linear product nerolidol (**S9**), and when co-expressed with GPPS, it produces the linear monoterpene linalool (**M2**) (Figs 2D and S4).

Overall, among the eight TPS gene family members, NnTPS4 and NnTPS10 from the TPS-a subfamily demonstrated substrate promiscuity, yielding a variety of products. Meanwhile, NnTPS9 (TPS-b) produces monoterpenes and NnTPS3 (TPS-g) generates linear terpene products.

### Residue N314 of NnTPS10 determines the secondary cyclization in γ-eudesmol production

**Figure 3:**
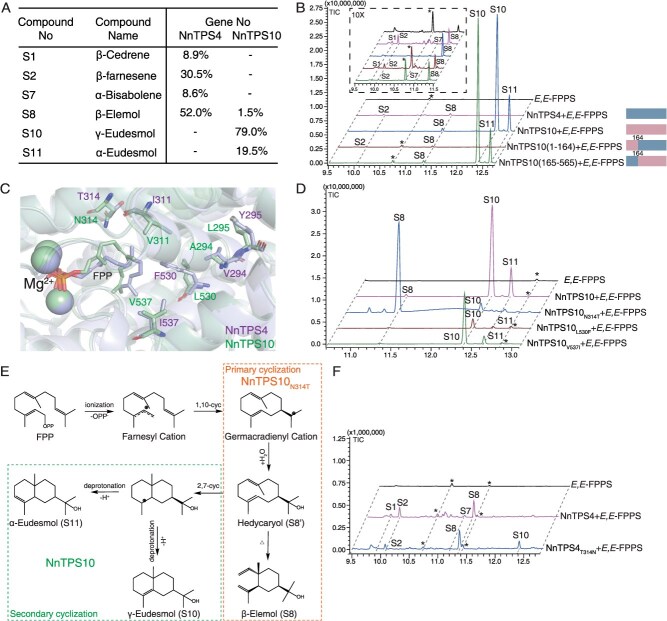
Residue N314 of NnTPS10 determines the secondary cyclization in γ-eudesmol production. (A) Comparison of terpene products produced by NnTPS4 and NnTPS10. (B) GC spectrum profile of enzymatic products from NnTPS4, NnTPS10, and their chimeric enzymes. (C) Molecular docking of predicted NnTPS4 and NnTPS10 structures with FPP substrate. (D) GC spectrum profileof enzymatic products from NnTPS10 and its mutants. (E) Proposed catalytic pathway for the biosynthesis of γ-eudesmol. (F) GC spectrum profile of enzymatic products from NnTPS4 and its mutant. Asterisk indicates unspecific products.

Despite sharing 94% similarity in their amino acid sequences, NnTPS10 and NnTPS4 exhibit significant differences in their catalytic products. Notably, only NnTPS10 produces γ-eudesmol (**S10**) (Figs 2B, 3A and B, and  S7). To further explore the catalytic mechanism, we created two chimeric enzymes by swapping amino acid segments between NnTPS4 and NnTPS10 (Fig. 3B). NnTPS10(1–164) was made by combining the N-terminal (residues 1–164) of NnTPS10 with the C-terminal (residues 165–565) of NnTPS4, while NnTPS10 (165–565) was constructed by fusing the N-terminal (residues 1–164) of NnTPS4 with the C-terminal (residues 165–565) of NnTPS10. The results showed that NnTPS10 (1–164) lost all activity, while NnTPS10 (165–565) produced a similar product profile (Fig. 3B), indicating that the 165–565 segment is crucial for NnTPS10 activity.

Subsequently, we conducted protein structure predictions for NnTPS4 and NnTPS10 by AlphaFold2 [38], and modelled the predicted NnTPS4 and NnTPS10 structure with FPP substrate equivalent to that observed in the crystal structure of 5-*epi*-aristolochene synthase with FPP (PDB:5IK0) [39] (Fig. 3C). A sequence alignment of residues 165–565 between NnTPS4 and NnTPS10 identified 25 divergent amino acids that may contribute to functional divergence in terpene synthase activity (Fig. S7). Among these, six residues—located at positions 294, 295, 311, 314, 530, and 537—were situated near the catalytic pocket (Fig. 3C). We then performed reciprocal single-point mutations at these six positions in NnTPS10 and NnTPS4.

While NnTPS10_V537I_ and NnTPS10_L530F_ resulted in varying degrees of decreased enzymatic activity, NnTPS10_N314T_ showed a pronounced reduction in the production of γ-eudesmol (**S10**) and α-eudesmol (**S11**). In contrast, the yields of β-elemol (**S8**) increased markedly (Fig. 3D). β-Elemol is likely the result of thermal rearrangements from hedycaryol, occurring during the heating process of gas chromatography–mass spectrometry (GC–MS) analysis; therefore, large-scale assays were conducted, and the products were ultimately verified as hedycaryol (**S8′**) through NMR analysis and comparison with the literature [40] (Figs S8 and S9). In the proposed biosynthesis pathway of γ-eudesmol (**S10**), hedycaryol (**S8′**) acts as a key intermediate during the 1,10-cyclization step. The NnTPS10_N314T_ mutation disrupts the 2,7-closure of hedycaryol (**S8′**), blocking the secondary cyclization required for γ-eudesmol (**S10**) formation. As a result, the accumulation of hedycaryol (**S8′**) significantly increases (Fig. 3E).

Consistent with these findings, the reciprocal mutation NnTPS4_T314N_ significantly reduced the production of β-elemol (**S8**), while enabling the synthesis of γ-eudesmol (**S10**) (Fig. 3F). These results demonstrate that N314 is a critical residue governing the 2,7-cyclization of the carbocation intermediate, which determines the bifurcation between hedycaryol (**S8′**) and γ-eudesmol (**S10**). Notably, the mutation NnTPS4_T314N_ completely eliminated its ability to produce diterpene cembrene A (Fig. S10), underscoring its essential role in stabilizing the extended carbocation intermediate necessary for macrocyclic diterpene formation.

### Γ-eudesmol demonstrates *in vitro* insecticidal and antimicrobial activities

**Figure 4:**
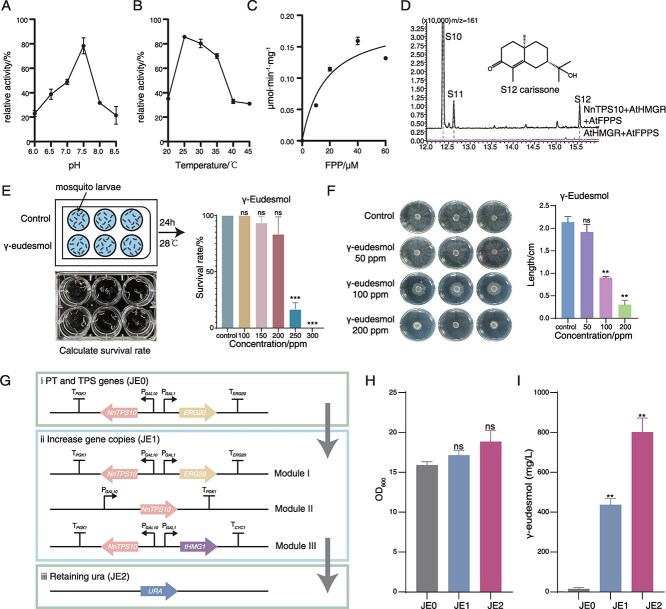
Insecticidal and antimicrobial activities of γ-eudesmol and its heterologous biosynthesis. (A) pH-dependent activity profile of NnTPS10. (B) Temperature optima for NnTPS10-catalyzed FPP cyclization. (C) Substrate saturation kinetics of NnTPS10 with FPP. (D) GC spectrum profile of enzymatic products from the co-expression of NnTPS10, AtHMGR (GeneID: P14891.1), and AtFPPS (GeneID: NP_193452.1) *via* transient expression in *Nicotiana benthamiana* leaves. Carissone (S12) was identified by comparison to the NIST17 library. (E) Larvicidal activity of γ-eudesmol was tested against 14-day-old mosquito larvae (*Aedes aegypti*), in a treatment of 24 hours (*N* = 3; ns, not significant; ^***^P < 0.0001 vs control). (F) The antifungal activity of γ-eudesmol was tested against the phytopathogen *Rhizoctonia solani* strain YWK196, in 72 hours of growth (*N* = 3; ns, not significant; ^**^P < 0.001 vs control). (G) Schematic diagram of metabolic engineering strategies in *S. cerevisiae* JCR27 strain. (H) OD_600_ of the engineered yeast strains in 72-hour fermentation. (I) Quantification of γ-Eudesmol production in engineered yeast strains. Statistical differences were determined by *t*-test (*N* = 3; ns, not significant; ^**^P < 0.001 vs JE0).

Given that NnTPS10 can produce a high level of γ-eudesmol, we purified the NnTPS10 protein from heterologous expression in *Escherichia coli* and determined its optimal catalytic conditions *in vitro* (Fig. S11). The results showed that the catalytic activity was highest under conditions of 25°C and pH = 7.5 (Fig. 4A and B). Under this condition, enzyme kinetics parameters were further determined as *K_m_* = 16.71 *μ*M, *K_cat_* = 0.2158 *s^−1^*, and *K_cat_*/*K_m_* = 0.0129 s^−1^·*μ*M (Fig. 4C).

To explore the biosynthetic application of NnTPS10 in plants, we transformed NnTPS10 into tobacco leaves and co-expressed it with AtHMGR and AtFPPS to augment the supply of the substrate FPP (Fig. S12). The results showed that high concentrations of γ-eudesmol (**S10**) and α-eudesmol (**S11**) were detected *in vivo*, along with the presence of its oxidized product, carissone (**S12**) (Figs 4D and S12). Therefore, NnTPS10 can be applied to plant genetic engineering and synthetic biology of synthesizing γ-eudesmol, and the previous researches indicated that carissone also possesses antibacterial activity against *Staphylococcus aureus*, *Escherichia coli*, and *Pseudomonas aeruginosa* [41].

Plant essential oils containing γ-eudesmol have been reported to exhibit strong insecticidal and antimicrobial properties [21, 23]. However, research on the bioactivity of γ-eudesmol as a single compound remains limited. To address this, we performed large-scale fermentation and purification of γ-eudesmol from a yeast strain expressing NnTPS10 for bioactivity testing. To evaluate the insecticidal activity of γ-eudesmol, mosquito larvae (*Aedes aegypti*) were exposed to solutions containing varying concentrations of γ-eudesmol, and mortality rates were recorded after 24 hours. At a concentration of 250 ppm, γ-eudesmol effectively killed most mosquito larvae, and at 300 ppm, it achieved 100% larval mortality (Fig. 4E). Additionally, *in vitro* antimicrobial tests against the phytopathogenic fungus *Rhizoctonia solani* showed that γ-eudesmol completely inhibited fungal growth at 200 ppm (Fig. 4F).

### Heterologous biosynthesis of γ-eudesmol in *Saccharomyces cerevisiae*

To establish heterologous γ-eudesmol biosynthesis in *Saccharomyces cerevisiae*, we chromosomally integrated *NnTPS10* and *ERG20* (FPPS) into the JCR27 strain, generating strain JE0, which produced γ-eudesmol at 15.52 mg/L (Fig. 4G–I). To substantially enhance γ-eudesmol production, we implemented systematic metabolic engineering strategies in this yeast chassis.

Clustered regularly interspaced short palindromic repeats (CRISPR) technology was used to integrate three additional copies of *NnTPS10* along with *ERG20* and *tHMG1* gene into the yeast chromosome (Fig. 4G; Table S7). Among these, tHMG1 serves as the key rate-limiting enzyme in the MVA pathway. The resulting recombinant strain was named JE1 (Fig. 4G). The γ-eudesmol titer in this strain reached 436.88 mg/L, highlighting that the expression level of the *NnTPS10* gene is a critical determinant for γ-eudesmol synthesis (Fig. 4H and I). To restore uracil prototrophy and enable glucose-inducible expression (thereby eliminating the need for costly galactose induction), we integrated the *URA3* gene into the *GAL80* locus (Fig. 4G). The resulting engineered strain, JE2, maintained comparable growth characteristics (as reflected by OD_600_) to the parental strain JE0 while achieving a remarkable γ-eudesmol titer of 801.66 mg/L in shake-flask cultures (Fig. 4H and I), representing the highest yield reported to date.

## Discussion

γ-Eudesmol is a major component of the essential oil from the *N. nucifera* flower [11]. It is also found in the essential oils of many plants, including *E. stipitata*, *T. dolichophyllum* and *S. brevibracteata* [21–23]. These essential oils have been reported to have insecticidal and antimicrobial activities. However, the specific role of γ-eudesmol in plant biotic stress responses has not been reported, due to the challenges and high costs with isolating it from essential oils. In this study, we synthesized the γ-eudesmol using synthetic biology methods, enabling the first investigation of its antimicrobial and larvicidal bioactivities (Fig. 4E and F). This finding suggests that γ-eudesmol may serve as a defense metabolite, protecting the sacred lotus from pathogenic infections and pest infestations in aquatic ecosystems. Further investigation through plant-based experiments, such as gene overexpression or gene knock-out, are needed to fully explore the additional biological activity.

Functional characterization of the two TPS-c subfamily genes (*NnTPS6* and *NnTPS7*) showed that they can exclusively produce *ent*-CPP, which was confirmed through rigorous chiral resolution (Fig. 1C). Their distinct expression patterns and significantly different catalytic activities suggest that they may mediate gibberellin biosynthesis, thereby regulating plant development. Some studies show that *N. nucifera* is able to produce (+)-CPP-derived diterpenes, such as pimaric acid [42]. From a genomic perspective, this seems unlikely, but different ecotypes of *N. nucifera* may have genomic variations, possibly containing atypical type-c genes capable of producing (+)-CPP.

The catalytic mechanisms underlying terpenoid cyclization currently remain incompletely understood. A previous study in 5-*epi*-aristolochene synthase showed that a catalytic triad D444-Y520-D525 facilitates proton transfer cascades to stabilize carbocation intermediates during secondary cyclization [39]. While this catalytic triad (D461-Y537-D541) was fully conserved in NnTPS4 and NnTPS10, our results demonstrated that the N314T mutation disrupted the second cyclization step required for γ-eudesmol formation (Fig. 3C–F). This observation suggests that N314 plays a critical role during the secondary cyclization. The precise mechanism by which this residue influences terpene synthase activity remains to be elucidated. Further structural studies (X-ray crystallography or molecular dynamics simulations) and mutagenesis analysis will be essential to fully unravel this novel regulatory mechanism.

Additionally, through the NnTPS10_N314T_ mutation, we obtained hedycaryol—a key intermediate in γ-eudesmol synthesis—with significant high yield (Fig. 3D). Hedycaryol has been identified in various plants, as well as in bacteria and fungi. Normally, it is reported as elemol due to the occurrence of Cope rearrangement during isolation. Both hedycaryol and elemol exhibit notable insecticidal activity and are used as fragrance ingredients [43, 44], highlighting their possibility for further development. Notably, hedycaryol is also a proposed biosynthetic intermediate for eudesmols and guaiols [45]. Thus, further protein engineering of NnTPS10 could enable the production of widespread sesquiterpene alcohols.

Through chromosomal integration of multiple *NnTPS10* gene copies combined with reinforcement of the MVA pathway *via* overexpression of rate-limiting enzymes (*HMG1* and *ERG20*), we achieved a dramatic improvement in γ-eudesmol biosynthesis (Fig. 4G). The engineered strain produced 801.66 mg/L γ-eudesmol, representing a 2.34-fold increase over the previously reported highest yield (342.32 mg/L) obtained from a recombinant yeast strain expressing CaTPS18 from *Celastrus angulatus* [46]. Furthermore, the strategic integration of *URA* into the *GAL80* locus enabled uracil prototrophy and eliminated the requirement for galactose induction during fermentation. The above modification not only significantly reduced production costs but also enhanced production yields (Fig. 4H and I). This effect may benefit from the improved cellular fitness as demonstrated in related metabolic engineering studies. To further enhance the production of γ-eudesmol, strategies such as knocking out *ERG9* to reduce competitive flux toward sterol biosynthesis, optimizing fed-batch fermentation conditions, or engineering NnTPS10 to improve enzyme performance can be implemented [47]. These approaches would further boost production yield while advancing toward industrial-scale manufacturing of γ-eudesmol.

## Materials and methods

### Plasmids and strains

The *S. cerevisiae* strain JCR27, previously characterized for terpenoid biosynthesis in prior studies [30–32], was utilized as the starting strain for all genetic constructs. Codon-optimized *N. nucifera* TPS genes, tailored for heterologous expression in *S. cerevisiae* and *E. coli*, were synthesized by GeneCreate Biological Engineering Co., Ltd. (Wuhan, China). Amplification reactions employed Phanta Max Super-Fidelity DNA Polymerase following the supplier’s specifications. Primers are listed in Table S5. Subsequently, amplified fragments underwent purification using the FastPure EndoFree Plasmid Mini Plus Kit. Plasmid assembly was performed *via* a modified LiAc/SS carrier DNA/polyethylene glycol protocol [30], with engineered strains and plasmid details cataloged in Table S6. The shake-flask fermentations were performed as previously reported [30, 48].

### Structural identification of terpenoid compounds

Following phase separation, the organic layer was harvested from the biphasic culture system, then diluted with hexane before analytical procedures. The structural identification of terpenoid compounds was rigorously confirmed through a battery of analytical methods, including GC–MS and NMR spectroscopy. The GC–MS and NMR spectroscopy analysis were performed as previously reported [31]. Compound identifications were achieved by matching retention times and diagnostic fragmentation patterns against authentic chemical standards or the NIST17 mass spectral reference database. For NMR, chemical shifts (*δ*, ppm) were calibrated against CDCl₃ (*δ*_H_ 7.26/*δ*_C_ 77.16) and (CD_3_)_2_CO (*δ*_H_ 2.05/*δ*_C_ 29.84/*δ*_C_ 206.26) as internal references.

### Isolation and purification of γ-eudesmol in yeast

Following large-scale fermentation of γ-eudesmol using *S. cerevisiae*, the cells were harvested and lysed *via* high-pressure homogenization. The resulting lysate underwent ethyl acetate extraction, whereupon the organic layer was isolated, concentrated *via* rotary evaporation under reduced pressure, and yielded crude extract. Subsequent purification employed silica gel column chromatography (500–800 mesh) with a stepwise polarity gradient (petroleum ether/ethyl acetate, 100:1 v/v). Eluted fractions were analyzed by thin-layer chromatography, pooled, and concentrated by rotary evaporation to yield γ-eudesmol. The purity of the final product was verified by GC–MS.

### Larvicidal activity assay against mosquito larvae

To evaluate the larvicidal efficacy of the test compound, a six-well plate-based bioassay was conducted. The compound was dissolved in dimethyl sulfoxide (DMSO) to prepare a stock solution (10 000 ppm). Each well received 10 ml of ddH₂O, followed by the addition of the stock solution to achieve desired test concentrations (i.e. 100, 150, 200 ppm). Control wells were treated with an equivalent volume of DMSO. Ten 10-day-old mosquito larvae were transferred into each well. After 24 hours of exposure at 25°C, mortality was assessed by gentle prodding with a pipette tip: larvae failing to respond to mechanical stimulation were recorded as dead. Three biological replicates were performed for statistical analysis. Statistical significance between groups was determined by Student’s *t*-test (^*^P < 0.01, ^**^P < 0.001 and ^***^P < 0.0001).

### Inhibition zone assay of *Rhizoctonia solani*

To determine the inhibition of test compound to *Rhizoctonia solani*, mycelial plugs (7 mm diameter) excised from the periphery of three-day-old colonies were inoculated onto potato dextrose agar (PDA, Beijing Solarbio Science & Technology Co., Ltd.) supplemented with specified concentrations of test compounds dissolved in DMSO. DMSO-only PDA plates served as negative controls. Following 24 hours static incubation at 28°C, radial colony growth was quantified *via* cross-diameter measurements, subtracting the initial plug size (7 mm). At least three replicates were examined for each experiment. Statistical significance between groups was determined by Student’s *t*-test (^*^P < 0.01, ^**^P < 0.001, and ^***^P < 0.0001).

## Supplementary Material

Web_Material_uhaf191

## Data Availability

Sequences of NnTPSs in this study were provided in Table S2.

## References

[1] David B, Wolfender J, Dias DA. The pharmaceutical industry and natural products: historical status and new trends. Phytochem Rev. 2015;14:299–315

[2] Cameron DE, Bashor CJ, Collins JJ. A brief history of synthetic biology. Nat Rev Microbiol. 2014;12:381–90 24686414 10.1038/nrmicro3239

[3] Keasling JD . Synthetic biology and the development of tools for metabolic engineering. Metab Eng. 2012;14:189–95 22314049 10.1016/j.ymben.2012.01.004

[4] Li Y, Qin W, Liu H. et al. Increased artemisinin production by promoting glandular secretory trichome formation and reconstructing the artemisinin biosynthetic pathway in *Artemisia annua*. Hortic Res. 2023;10:uhad55 10.1093/hr/uhad055 PMC10199714 37213685

[5] Du B, Sun M, Hui W. et al. Recent advances on key enzymes of microbial origin in the lycopene biosynthesis pathway. J Agric Food Chem. 2023;71:12927–42 37609695 10.1021/acs.jafc.3c03942

[6] Li Y, Svetlana P, Yao J. et al. A review on the taxonomic, evolutionary and phytogeographic studies of the lotus plant (Nelumbonaceae: *Nelumbo*). Acta Geol Sin. 2014;88:1252–61

[7] Qi H, Yu F, Deng J. et al. Studies on lotus genomics and the contribution to its breeding. Int J Mol Sci. 2022;23:7270 35806274 10.3390/ijms23137270 PMC9266308

[8] Li F, Sun XY, Li XW. et al. Enrichment and separation of quercetin-3-O-β-d-glucuronide from lotus leaves (*nelumbo nucifera* gaertn.) and evaluation of its anti-inflammatory effect. J Chromatogr B. 2017;1040:186–91 10.1016/j.jchromb.2016.12.017 27987489

[9] Rai S, Wahile A, Mukherjee K. et al. Antioxidant activity of *Nelumbo nucifera* (sacred lotus) seeds. J Ethnopharmacol. 2006;104:322–7 16239089 10.1016/j.jep.2005.09.025

[10] Huang B, Ban X, He J. et al. Comparative analysis of essential oil components and antioxidant activity of extracts of *Nelumbo nucifera* from various areas of China. J Agric Food Chem. 2010;58:441–8 19919095 10.1021/jf902643e

[11] Zhang C, Guo M. Comparing three different extraction techniques on essential oil profiles of cultivated and wild lotus (*Nelumbo nucifera*) flower. Life. 2020;10:209 32948021 10.3390/life10090209 PMC7555187

[12] Singulani JL, Pedroso RS, Ribeiro AB. et al. Geraniol and linalool anticandidal activity, genotoxic potential and embryotoxic effect on zebrafish. Future Microbiol. 2018;13:1637–46 30480459 10.2217/fmb-2018-0200

[13] Chaudhary SC, Alam MS, Siddiqui MS. et al. Perillyl alcohol attenuates Ras-ERK signaling to inhibit murine skin inflammation and tumorigenesis. Chem Biol Interact. 2009;179:145–53 19161993 10.1016/j.cbi.2008.12.016

[14] Zhou F, Pichersky E. More is better: the diversity of terpene metabolism in plants. Curr Opin Plant Biol. 2020;55:1–10 32088555 10.1016/j.pbi.2020.01.005

[15] Zhou F, Pichersky E. The complete functional characterisation of the terpene synthase family in tomato. New Phytol. 2020;226:1341–60 31943222 10.1111/nph.16431 PMC7422722

[16] Akhtar TA, Matsuba Y, Schauvinhold I. et al. The tomato cis-prenyltransferase gene family. Plant J. 2013;73:640–52 23134568 10.1111/tpj.12063

[17] Schilmiller AL, Schauvinhold I, Larson M. et al. Monoterpenes in the glandular trichomes of tomato are synthesized from a neryl diphosphate precursor rather than geranyl diphosphate. Proc Natl Acad Sci USA. 2009;106:10865–70 19487664 10.1073/pnas.0904113106 PMC2705607

[18] Baghban R, Farajnia S, Rajabibazl M. et al. Yeast expression systems: overview and recent advances. Mol Biotechnol. 2019;61:365–84 30805909 10.1007/s12033-019-00164-8

[19] Guo S, Wang D, Yang T-T. et al. Construction of cell factories for production of patchoulol in *Saccharomyces cerevisiae*. Zhongguo Zhong Yao Za Zhi. 2023;48:2316 37282860 10.19540/j.cnki.cjcmm.20230213.104

[20] Ye Z, Huang Y, Shi B. et al. Coupling cell growth and biochemical pathway induction in *Saccharomyces cerevisiae* for production of (+)-valencene and its chemical conversion to (+)-nootkatone. Metab Eng. 2022;72:107–15 35296429 10.1016/j.ymben.2022.03.005

[21] Dos Santos CRB, Sampaio MGV, Vandesmet LCS. et al. Chemical composition and biological activities of the essential oil from *Eugenia stipitata* McVaugh leaves. Nat Prod Res. 2023;37:3844–50 36469681 10.1080/14786419.2022.2151008

[22] Kart , Günal B, Mutlu D. et al. Evaluating antibiofilm, cytotoxic and apoptotic activities of *Scutellaria brevibracteata* subsp. *Brevibracteata* essential oil. Chem Biodivers. 2023;20:e202300878 37947368 10.1002/cbdv.202300878

[23] Nitwal L, Palni M, Melkani AB. et al. *Tanacetum dolichophyllum* (Kitam.) Kitam: chemical composition, variation, and antibacterial activity of essential oil from flowers, leaves and roots. Journal of Essential Oil-Bearing Plants. 2023;26:493–501

[24] Bomfim DS, Ferraz RPC, Carvalho NC. et al. Eudesmol isomers induce caspase-mediated apoptosis in human hepatocellular carcinoma HepG2 cells. Basic Clin Pharmacol Toxicol. 2013;113:300–6 23786320 10.1111/bcpt.12097

[25] Wang K, Deng J, Damaris RN. et al. LOTUS-DB: an integrative and interactive database for *Nelumbo nucifera* study. Database. 2015;2015:bav23 10.1093/database/bav023 PMC4383347 25819075

[26] Tholl D, Lee S. Terpene specialized metabolism in *Arabidopsis thaliana*. Arabidopsis Book. 2011;9:e143 10.1199/tab.0143 PMC3268506 22303268

[27] Sun Y, Zhang PT, Kou DR. et al. Terpene synthases in rice pan-genome and their responses to *Chilo suppressalis* larvae infesting. Front Plant Sci. 2022;13:905982 35668795 10.3389/fpls.2022.905982 PMC9164016

[28] Martin DM, Aubourg S, Schouwey MB. et al. Functional annotation, genome organization and phylogeny of the grapevine (*Vitis vinifera*) terpene synthase gene family based on genome assembly, FLcDNA cloning, and enzyme assays. BMC Plant Biol. 2010;10:226 20964856 10.1186/1471-2229-10-226 PMC3017849

[29] Falara V, Akhtar TA, Nguyen TTH. et al. The tomato terpene synthase gene family. Plant Physiol. 2011;157:770–89 21813655 10.1104/pp.111.179648 PMC3192577

[30] Siemon T, Wang Z, Bian G. et al. Semisynthesis of plant-derived englerin a enabled by microbe engineering of guaia-6,10(14)-diene as building block. J Am Chem Soc. 2020;142:2760–5 31999448 10.1021/jacs.9b12940

[31] Zhi Y, Dai C, Fang X. et al. Gene-directed in vitro mining uncovers the insect-repellent constituent from mugwort (*Artemisia argyi)*. J Am Chem Soc. 2024;146:30883–92 39485326 10.1021/jacs.4c08857

[32] Cheng W, Zhi Y, Chen F. et al. Characterization and functional reconstruction of a highly productive germacrene a synthase from *Liriodendron chinense*. Plant Biotechnol J. 2025;23:1927–37 40011225 10.1111/pbi.70023 PMC12120886

[33] Tholl D . Biosynthesis and biological functions of terpenoids in plants. Adv Biochem Eng Biotechnol. 2015;148:63 25583224 10.1007/10_2014_295

[34] Pelot KA, Chen R, Hagelthorn DM. et al. Functional diversity of diterpene synthases in the biofuel crop switchgrass. Plant Physiol. 2018;178:54–71 30008447 10.1104/pp.18.00590 PMC6130043

[35] Zhou K, Gao Y, Hoy JA. et al. Insights into diterpene cyclization from structure of bifunctional abietadiene synthase from *Abies grandis*. J Biol Chem. 2012;287:6840–50 22219188 10.1074/jbc.M111.337592 PMC3307272

[36] Liang J, Shen Q, Wang L. et al. Rice contains a biosynthetic gene cluster associated with production of the casbane-type diterpenoid phytoalexin *ent*-10-oxodepressin. New Phytol. 2021;231:85–93 33892515 10.1111/nph.17406 PMC9044444

[37] van Beilen JB, Holtackers Ŕ, Lüscher D. et al. Biocatalytic production of perillyl alcohol from limonene by using a novel mycobacterium sp. cytochrome P450 alkane hydroxylase expressed in *pseudomonas putida*. Appl Environ Microbiol. 2005;71:1737–44 15811996 10.1128/AEM.71.4.1737-1744.2005 PMC1082528

[38] Varadi M, Anyango S, Deshpande M. et al. AlphaFold protein structure database: massively expanding the structural coverage of protein-sequence space with high-accuracy models. Nucleic Acids Res. 2022;50:D439–44 34791371 10.1093/nar/gkab1061 PMC8728224

[39] Starks CM, Back K, Chappell J. et al. Structural basis for cyclic terpene biosynthesis by tobacco 5-*epi*-aristolochene synthase. Science. 1997;277:1815–20 9295271 10.1126/science.277.5333.1815

[40] Xu H, Lackus ND, Kollner TG. et al. Isotopic labeling experiments solve the hedycaryol problem. Org Lett. 2022;24:587–91 34985289 10.1021/acs.orglett.1c04021

[41] Lindsay EA, Berry Y, Jamie JF. et al. Antibacterial compounds from *Carissa lanceolata* R. Br. Phytochemistry. 2000;55:403–6 11140600 10.1016/s0031-9422(00)00343-5

[42] Qin L, du F, Yang N. et al. Transcriptome analyses revealed the key metabolic genes and transcription factors involved in terpenoid biosynthesis in sacred lotus. Molecules. 2022;27:4599 35889471 10.3390/molecules27144599 PMC9320166

[43] Cheng SS, Lin CY, Chung MJ. et al. Chemical composition and antitermitic activity against *Coptotermes formosanus* S_HIRAKI_ of *Cryptomeria japonica* leaf essential oil. Chem Biodivers. 2012;9:352–8 22344910 10.1002/cbdv.201100243

[44] Bhatia SP, Letizia CS, Api AM. Fragrance material review on elemol. Food Chem Toxicol. 2008;46:S147–8 18640176 10.1016/j.fct.2008.06.045

[45] Xu H, Dickschat JS. Hedycaryol - central intermediates in sesquiterpene biosynthesis, part II. Chemistry. 2022;28:e202200405 35239190 10.1002/chem.202200405 PMC9310801

[46] Li W . Genome Mining and Heterologous Efficient Synthesis of Celangulin in Saccharomyces Cerevisiae PhD dissertation. Tianjin University, Tianjing, China 2021

[47] Li M, Chen R, Qiao J. et al. Recent advances in multiple strategies for the biosynthesis of sesquiterpenols. Biomolecules. 2025;15:664 40427558 10.3390/biom15050664 PMC12108891

[48] Huang Y, Ye Z, Wan X. et al. Systematic mining and evaluation of the sesquiterpene skeletons as high energy aviation fuel molecules. Adv Sci. 2023;10:e2300889 10.1002/advs.202300889 PMC10427387 37271925

