# Supplementary material for: Systematic identification of terpene synthases from sacred lotus (Nelumbo nucifera) and heterologous biosynthesis of the insecticidal and antimicrobial compound γ-eudesmol
Source: Hortic Res. 2025 Jul 28;12(10):uhaf191. doi: 10.1093/hr/uhaf191 (PMC12539867; doi:10.1093/hr/uhaf191)
Supplement: Web_Material_uhaf191 [file web_material_uhaf191.docx]

Supplementary Information for

**­Systematic identification of terpene synthases from sacred lotus (*Nelumbo nucifera*) and heterologous biosynthesis of the insecticidal and antimicrobial compound γ-eudesmol**

Zhenni Xu^1,2^, Xueting Fang^1,2^, Yao Zhi^1,3^, Xiaochun Xiao^1,2,4^, Jing Yang^1,2^, Jie Hu^1,2^, Hangzhi Zhu^1,2^, Fangfang Chen^1,2^, Weijia Cheng^1,5^, Tiangang Liu^3,4^, Li Lu^1,2*^

^1^Department of Urology, Zhongnan Hospital of Wuhan University, Hubei Provincial Research Center for Basic Biological Science, School of Pharmaceutical Sciences, Wuhan University, Wuhan 430071, China;

^2^State Key Laboratory of Hybrid Rice, Hubei Hongshan Laboratory, School of Pharmaceutical Sciences, Wuhan University, Wuhan 430071, China;

^3^Wuhan Hesheng Technology Co., Ltd. Wuhan 430074, China;

^4^School of Life Sciences and Biotechnology, Shanghai Jiao Tong University, 200030, Shanghai, China;

^5^Department of Pharmacy, Renmin Hospital of Wuhan University, Wuhan 430060, China

^*^ Corresponding author: luliwhu@whu.edu.cn (L. Lu)

[2022203060020@whu.edu.com](mailto:1902358672@qq.com) (Z. Xu) [2021203060042@whu.edu.cn](mailto:2021203060042@whu.edu.cn) (X. Fang)

zhiyao@heshengtech.com.cn (Y. Zhi) [2019303060013@whu.edu.cn](mailto:2019303060013@whu.edu.cn) (X. Xiao)

[2022203060050@whu.edu.cn](mailto:2022203060050@whu.edu.cn) (J. Yang) 2023203060019@whu.edu.cn (J. Hu)

zhuhangzhi@whu.edu.cn (H. Zhu) [2018303060089@whu.edu.cn](mailto:2018303060089@whu.edu.cn) (F. Chen)

[HT005680@whu.edu.cn](mailto:HT005680@whu.edu.cn) (W. Cheng) liutg@whu.edu.cn (T. Liu)

**Supplementary Materials and Methods**

**Plant materials, chemicals and reagents**

*Nelumbo nucifera* “Jianxuan 17” was obtained from the Wuhan Botanical Garden (30.54°N, 114.39°E). The fresh and healthy leaf, petal, and seed tissues were immediately flash-frozen in liquid nitrogen for RNA extraction. Tryptone and yeast extract were purchased from Oxoid (Hampshire, UK). NaCl, glucose, galactose, components of the trace metal solution, and vitamin solution were purchased from Aladdin Biochemical Technology Co., Ltd. (Shanghai, China). Yeast nitrogen base (YBN), amino acids, ampicillin, synthetic dropout media, and Yeast extract peptone dextrose (YPD) media were purchased from Coolaber (Beijing, China). Farnesyl pyrophosphate ammonium salt was purchased from Sigma-Aldrich (St. Louis, MO, USA). All solvents in this study were analytical reagent grade from Sangon Biotech Co., Ltd. (Shanghai, China). Phanta Max Super-Fidelity DNA Polymerase and FastPure EndoFree Plasmid Mini Plus Kit were purchased from Vazyme Biotech Co., Ltd. (Nanjing, China). All primers used in polymerase chain reaction (PCR) were synthesized commercially by AuGCT Biotech Co., Ltd. (Wuhan, China) and Sangon Biotech Co., Ltd. (Shanghai, China).

**RNA extraction and quantitative real-time PCR**

Total RNA was extracted from plant materials using the FastPure Plant Total RNA Isolation Kit (Vazyme Biotech Co., Ltd., Nanjing, China) according to the manufacturer's instructions. One microgram of RNA was reverse transcribed into cDNA with SuperScript III (ThermoFisher) followed by a quantitative PCR assay with SYBR Green Master Mix using CFX96 Real-Time System 690 (Bio-Rad). Primers used in this study are listed in Table S5.

**Identification and phylogenetic analysis**

The protein sequences of *N. nucifera* were downloaded from the LOTUS-DB database (http://lotus-db.wbgcas.cn, accessed on 28 December 2021)^1^. To identify putative TPS genes, all predicted amino acid sequences were systematically scanned against the Pfam database (v28.0) using HMMER. A dual-domain filtering strategy was applied: sequences simultaneously harboring the terpene_synth_C domain (PF03936) and the terpene_synth_N domain (PF01397) were retained, with an E-value cutoff of 1e-5 to ensure stringent homology detection. For phylogenetic reconstruction, full-length amino acid sequences of the candidate TPS genes were aligned using MAFFT (v7.475)^2^. The phylogenetic tree was constructed with FastTree (v2.1.11) under the maximum-likelihood method with 1,000 bootstrap replicates^3^.

**GC-MS analysis**

Sample aliquots (1 μL) were injected into a TQ8040 triple quadrupole mass spectrometer (Shimadzu, Kyoto, Japan) equipped with an SH-Rxi-5Sil MS capillary column (30 m × 0.25 mm × 0.25 μm). The GC oven temperature program was initiated at 80°C for 1 min, followed by a linear gradient of 10°C·min^-1^ to 280°C, with a final isothermal phase of 7 min. Mass spectral data were acquired in full-scan mode across a mass-to-charge ratio (*m/z*) range of 45 to 500.

**Enzymatic assays**

The candidate genes were cloned into the pET-28a(+) vector for heterologous expression in *E. coli* strain BL21(DE3)pLysS. Cells were lysed *via* sonication, and the supernatant was purified by Ni-NTA affinity chromatography under native conditions, as described previously^4^.

NnTPS10 enzyme activity reaction was conducted in a total reaction volume of 50 μL consisting of 50 mM Tris buffer (pH 7.0), 5 mM MgCl_2_, 5 mM KCl, 2 mM DTT and 10% glycerol. 50 μM FPP and 5 μg/mL purified protein were incubated at 30°C for 1 hour. 100 μL of chromatographic grade ethyl acetate was added, and the mixture was extracted with shaking at 800 × g for 15 min. After centrifugation, the upper organic phase was removed for GC-MS detection. To determine the optimal enzymatic reaction temperature, the mixture was maintained at pH 7.0 across a temperature gradient (20, 25, 30, 35, 40, and 45°C). For pH dependency studies at 30°C, three buffering systems were employed: Na_2_HPO_4_-CiH_3_ buffer (30 mM, pH 5.5–6.0), NaH_2_PO_4_-Na_2_HPO_4_ buffer (30 mM, pH 6.5–7.5) and glycine-NaOH buffer (30 mM, pH 8.0-8.5). For enzyme reaction kinetics determination, serial dilutions of farnesyl diphosphate (10-50 μM) and 5 μg/mL purified protein were added to the reaction at the optimal pH and reaction temperature, and the reaction was carried out for 15 min. The enzyme reaction kinetics curve was fitted using GraphPad Prism 9.3.1 to calculate *Km* and *Kcat* values.

**Molecular docking**

The protein structure of NnTPS4 and NnTPS10 was predicted using AlphaFold^5^. The Mg²⁺ coordination shell (comprising a PPi group and three Mg²⁺ ions) was reconstructed based on the conserved coordination motif of terpene synthases (PDB ID: 5IK0)^6^. In PyMOL (http://www.pymol.org/pymol), the farnesyl chain was manually modeled into the active site cavity following the method reported before^7^, ensuring avoidance of steric clashes with surrounding residues and positioning in a cyclization-ready conformation (dC1-C10 < 5 Å). Molecular docking simulations were subsequently performed using Discovery Studio (version 19.1.0).

***Nicotiana benthamiana* transient expression**

The vector pEAQ-HT was used for transient expression in *N. benthamiana*, and the procedure was done as reported previously^8^. Primers used in the construction of the vectors are listed in Table S5. pEAQ-HT constructs carrying specific genes were mobilized into *Agrobacterium tumefaciens* strain GV3101. *A. tumefaciens* cells were incubated in liquid Luria-Bertani medium and grown overnight to an OD_600_ value of 1, then centrifuged at 5,000 × g for 10 min to remove the Luria-Bertani medium. The cells were resuspended in an equal volume of MMA buffer (10 mM MES, pH 6.8, 10 mM MgCl_2_, and 100 μM acetosyringone) to an OD_600_ of 1.0 and 2-4 hours incubation at ambient temperature. Cell suspensions were injected into leaves of 4-week-old *N. benthamiana* from the abaxial side using a 3 mL syringe. The infiltrated leaves were collected 4 days after infiltration.

To analyze the compounds produced in the leaves, the harvested plant materials were flash frozen and ground into a fine powder in liquid nitrogen. 300 mg of ground leaf tissues were extracted with 600 μL MTBE. The extracts were incubated by ultrasound extraction pretreatment for 40 min, followed by centrifugation for 5 min at 13,000 × g. The MTBE layer was transferred to a fresh vial, dehydrated using anhydrous Na_2_SO_4_, and concentrated by evaporating the solvent to a final volume of about 300 μL. Analysis of the samples was performed with an Rxi-5Sil column on a Shimadzu QP-2010 GC-MS system.

**Yeast metabolic engineering for the production of γ-Eudesmol**

Various gene fragments were PCR-amplified for subsequent assembly. A fragment harboring *NnTPS10*, *ERG20*, the homologous arm of the *LEU* loci, and the *LEU* selective marker was amplified from plasmid pYJ900. Additionally, a fragment containing *NnTPS10*, the homologous arm of the *YPRCdelta15* loci, and the *TRP* selective marker was obtained from pYJ908. Similarly, a fragment containing *NnTPS10*, *tHMG1*, the homologous arm of the *URA3* loci, and the *HIS* selective marker was obtained from pYJ905. A CRISPR-Cas9-mediated multiplex integration strategy was employed to insert three additional *NnTPS10* expression cassettes into the genomic loci ChrX-4, XII-5, and XI-1. The preparation of double-stranded DNA (dsDNA) donors for homology-directed repair (HDR) was adapted from previous work^9^, with Cas9 target sites specified in Table S7. To enable *GAL80* knockout and restoration of the auxotrophic marker *URA3,* the homologous arm of the *GAL80* locus was fused to the *URA3* selective marker via overlap extension PCR (OE-PCR). All amplified fragments served as templates for homologous recombination-based genome editing.

**Reference**

1 Wang, K. *et al.* LOTUS-DB: an integrative and interactive database for *Nelumbo nucifera* study. *Database : The Journal of Biological Databases and Curation*. **2015**, bav23 (2015).

2 Katoh, K. & Standley, D. M. MAFFT multiple sequence alignment software version 7: improvements in performance and usability. *Mol Biol Evol*. **30**, 772 (2013).

3 Price, M. N., Dehal, P. S. & Arkin, A. P. FastTree 2--approximately maximum-likelihood trees for large alignments. *Plos One*. **5**, e9490 (2010).

4 Cheng, W. *et al.* Characterization and functional reconstruction of a highly productive germacrene A synthase from *Liriodendron chinense*. *Plant Biotechnol J.* (2025).

5 Varadi, M. *et al.* AlphaFold Protein Structure Database: massively expanding the structural coverage of protein-sequence space with high-accuracy models. *Nucleic Acids Res*. **50**, D439 (2022).

6 Starks, C. M., Back, K., Chappell, J. & Noel, J. P. Structural basis for cyclic terpene biosynthesis by tobacco 5-*epi*-aristolochene synthase. *Science*. **277**, 1815 (1997).

7 O Brien, T. E., Bertolani, S. J., Zhang, Y., Siegel, J. B. & Tantillo, D. J. Predicting productive binding modes for substrates and carbocation intermediates in terpene synthases bornyl diphosphate synthase as a representative case. *Acs Catal.* **8**, 3322 (2018).

8 Xu, H. *et al.* Coexpression analysis identifies two oxidoreductases involved in the biosynthesis of the monoterpene acid moiety of natural pyrethrin insecticides in *Tanacetum cinerariifolium*. *Plant Physiol*. **176**, 524 (2018).

9 Zhang, Y. *et al.* A gRNA-tRNA array for CRISPR-Cas9 based rapid multiplexed genome editing in *Saccharomyces cerevisiae*. *Nat. Commun.* **10**, 1010 (2019).

**Supplementary Tables**

**Table S1. Overview of *Nelumbo nucifera* terpene synthase (TPS) candidate genes.**

| **Gene** | **Name** | **DNA Length (bp)** | **Signal peptide** |
| --- | --- | --- | --- |
| *NnTPS1* | *Nnu11096* | 1341 | N.A. |
| *NnTPS2* | *Nnu16497* | 1809 | N.A. |
| *NnTPS3* | *Nnu03893* | 1827 | N.A. |
| *NnTPS4* | *Nnu01123* | 1695 | N.A. |
| *NnTPS5* | *Nnu00765* | 2436 | N.A. |
| *NnTPS6* | *Nnu06733* | 2487 | N.A. |
| *NnTPS7* | *Nnu06226* | 2373 | N.A. |
| *NnTPS8* | *Nnu10623* | 1833 | N.A. |
| *NnTPS9* | *Nnu20041* | 1791 | N.A. |
| *NnTPS10* | *Nnu08911* | 1692 | N.A. |
| *NnTPS11* | *Nnu22870* | 1686 | N.A. |

N. A., not avaibable.

**Table S2. Sequence information of *N.* *nucifera* TPS candidate genes.**

| **Gene** | **Sequence** |
| --- | --- |
| *NnTPS1* | MSANSFFIPPSSLSTKQFSQGSCLHPARPVLQHAQKVTKHNIQFLVVSSQFLNQEVIRRSGNYKPSSWDYDGLQSLKSEFLEEAFVERAKKLMVDVKRKLNQAAGAVDQLELIDTLQRLGVGYHFQSDIKSVLESIKDTEEELYAQQFTYRHLIKQLKEKTFPNYKNMARLVSHALEMPLHWRMKRWEARWFIDTYSMKQDVDPLILEFAKLDFNMVQAIYQQELKDASRWWTDLGLAEALRFSRDRIAESYLWTTGVIFEPQFGYCRRQLTKVNCLITTIDDVYGTLDELKLFTDTVERWDINRMGDLPDYMKMCFMALYNSINEMAYDVLKKSGRDILKYLKKGDELKRGDVPKSIQCYMRETGAPEEIAREHIRGLRAKEWKKMNKCMPAPSPFPRAFKDSAIDLGRMAQFMYQHGDGHGVLADCLTRNNIMSLLVNPIPLDKL |
| *NnTPS2* | MALKLNVMTPPSFAQLGSTSTRYVVARTTPSSHRPTWRIRCVANVQTQEDSEAARKAGTYTPTIWDYDFVESLKSDYASETYATRADKLKQDVRSRLVVTVDPLARLKLIDVLERLGVGYLFEKEIQEALQAVYSESSKQIMKEDLYATALHFRILRQHGHEVPQDVFNEFIDEKGKFKESLSQDVQGLLSLYEASYLGAEGESILDEAREFTKGHLKHLKENVDSRLAKQLSHALEIPLQWRMLRPENRWFIEVYEEQEDMDPTVLELAKLDFNMVQAVHQRDLRTASRWWRNLGLAQKLSFARDRLVESFLWSAGVTYEPQYRRCRNWLTKVMKFVLVLDDIYDVYGSLEELEIFTDAVERWDTAAMAQLPDYMKMSFLALFNTTNEMAYEILKEQGWDILPYLRKAWAGFIKAMFQEAKWYHKNSTPPVEDYLKNGWVSSSGTVFLVHAFFATGQEINKDVLDYLENTPDLIYGPSMIFRLSNDLATSSAELERGDVSSSIHCYMVQHNASEQVAREYIRGLTLDVWKKMNKSISNSPFSLQFVNFCLNLARTSLCIYQYGDGLGAESNKSKDHVLSLIIQPLGIESHKAPVNGSFLSVS |
| *NnTPS3* | MSFLSIITAEPTVQPITRTKLVEQWPSHGPKPIRWWNCRLHASQTTRNQSSLKLSTIFGPAEAFNIITCKASSGEFMTEKGSLLSWESPSALHMEDIICGKHRNRCEELCGLLLKVEDPLESMALIDALQRLCIDHHFEEEIRTVLSVLYTRFGSDIYQMGTGSLHDVSLCFRLFRQAGYCVPTDVFDKFRDNRGRFKPELNEDTRGMLSLFEASHLGIHGEDILDEANELAYKHLSASRLNLSPPLARAVEDTLKHPFNKSLASFKVKSYLKNFSSSYWWHNSLQELAKVEFNLVQSLHRQEVAQVSQWWRDLGLSDELKFARNQPLKWYMWSMVVLRDPRFSEQRIDLTKPIALIYIIDDIFDVYGTLDELVIFTEVINKWELVAAEQLPKYMRKCFKALYGITNEICTKVFKETGWNPINSLQKAWASLCNAFLVEAKWFSSRQLPNETDYLKNGIVSSGVHVALIHIFFLLGQGITKESVDYLDAAPAIISSSASILRLWDDLGSAKDENQEGYDGSYIECYMKNHQGISIEGARNHVLGMISDSWKQLNQECLSPNPFSPTFIEAIHNFAKMVSVMYDYDDYRRLPVLEEYINSLLFENIPLRG |
| *NnTPS4* | MSSQISSCPPTQHSSSEAEKTELIRHTTTFHPSIWGDRFITYTCDNMVKLESYNQQVEELKEEVRSILRNSANKPLEMLSLIDGVQRLGLGYQFEREIDKMLEQIFDAHIVYFNDMDDNLNAVALLFRLLRQHGYNIPCDVFKKFRDEDGKFRESLARDIQGMLSLYEAAYLGKRGEEVLDEALAFTTAHLKSTVATDTTSPTLVKQVKHALEQPLHRGIPKIEARHYISFYEEDKSRIQVLLKLAKLDFNRTQLLYLNELSQVSRWWKNIDFTSKTPYARDRLVECYFWSAGVYPEPQYSLGRKILAKIIAITTILDDTYDAYGTFEELQLLTDAIRRWDANETDKLPEYMKVIYVTLLNIYNEIEEEMRKEGRCFCVSYAKEAMKDLIEAYQIEATWLKEGHIPTFEEFSKLGLITSTCIMMTNVIFACMGEMATKEVFDWLSKTPDAVKSTCIIGRFLDDIQTNEFEQERGHVCSSIECYMKQYGVSSEEAKNELRKLVTNAWKVLNEECITPTAAPMPLLMPLVNFTRLADVIYKHGDAFTNVITGLKTHIKLLLIDPITI |
| *NnTPS5* | MSLIYPNNLLLKKPQRWQNSTLSVPDIYGTKTGGRASNATMCSHQPKERIRELFTKIELSVSAYDTAWVAMVPSPNSPQSPYFPECVNWLLENQLPDGSWCLPHSHPLLIKDALSSTLACVLALKRWNIGTNHIEKGIHFIVSNFPSSTDEKQHAPIGFDITFPGMIEYAREMGLVLPLSSTIADALFRKRNLEFRRTSESNSEGKKAYLAYIAEGLQNLQDWKEVMKYQRKNGSLFNSPSTTAAAFTHLQDANCLGYLRTVLKEFGGAVPTTYPLDIHAQLCMIDSLIGLGIDRHFRNEIKNVLDETYRCWLQKDERIFLDLDVCAMAFRILRTNGYNIAADALPQFVEEKHFLDSLGGHLNDIHVVLELYKASQFMIFPNEPVLEKHQFWLSWYLKQEISKCLENRDGVSRYITQEVDYALKFPHYANLERLENRRNMEHYNLNHPRILKTSYRCLNIHNIEFLELAIEDFNNCQEIYHKELKELERWVNDNKLDQLKFARQKLTYCYFSAAATLFSPELSDARISWAKNSLLTTVVDDFFDIGGSKEELENLIKLVEKWDENFAIGRCSEQVEIVFSAIHSTVNEIGDKAFKCQRRCVTNHLVEIWLNLLNSMMREADWLRNKSVPTMDEYMENGYVSFALGPIVLPALYLVGDTLSEEAVRSPEYHNLYKLMSTCGRLLNDIQGFEREGKQGKLNGVSLQMIHGSGVVKEEQVIREMRSLIENTRKELLGLVLQTNGSLVPRACKDLFWKMSKVLHLFYSKNDGFTSPHDMMRYVNEVIYEPFDPLRLEKMKISEVAAEECTGLLSSMDYKDD |
| *NnTPS6* | MPNTTAFLRLLPPVPVSRTYPSSDPGAVLSGVCSFATENKRSGFGLLRSRRNAIPRISAHVYTEILQNGLPTAEWLETLKNDREGEAPKVSISKEIVERVDSIKAMLSSMEDGEISISAYDTAWVSLVQDILGGGAPQFPSSLLWIVDNQLPDGSWGDHRIFSAHDRIISTLACVIALKSWNICPRKCDKGVVFIRENMSRLESENPEHMSIGFEVAFPSLIEIARKLHLQVPVDSTVMQMISAKRNLKLTRIPKEMMHIVPTTLLHSLEGMPGLDWEKLLKLQSPDGSFLFSPSSTAFALMQTKDENCLKYLKRVVERFNGGVPNVYPVDLFEHIWAVDRLERLGISRYFQSEIKECLDYVYRYWTEDGICWARNSTVHDIDDTAMAFRLLRLHGHDVSPDAFRHFEKGGEFFCFAGQSNQAITGMFNLYRASQVLFPGEKILEEAKTFSSRFLSEKQASNQLLDKWIITKDLPGEVRYALDIPWYASLPRLEARYYLEQYGGEDDVWIGKTLYRMPLVNNNVYLELAKLDFNNCQALHQHEWVNLQKWYTDCNLGEFGVNRGTLLQAYYVAAASIFEPERWTERLAWARTAVLVEAVSLYLEKEDPQRGAFVHDFFSNIGGSSIFSSDNKNKLDKRRWGSKRTAERLVEALLGTLNRLSLDSLLAHGQDVLLHLRRAWATWLLISLDEAEEEEEEEDKQQTGREAELLVRTINLCAGRPLSEELLCHPHYRRLVQLTNRVCHHLRRFREWKVNERSSDDPNTSSITTCEIESDMQELVQCVLRSSDGIDPAIKQTFLAVAKSYYYTAHCPPAMINLHIAKVLFESVH |
| *NnTPS7* | MIRTMNERNNLGLFHSILGAKSKSPTQVYAEVSKHGSAVIEWLETVNSDEEGEAHNVLISKEIAERVDSIKSMLNSMEDGEISISAYDTAWVALVEDIHGSGVPQFPSSLGWIVENQLPDGSWGDLGMFSAHDRILNTLACVVALKSWNVYPERWEKGMLFIRENMCMLETENAENMPIGFEIAFPSLIEIAKNLDLQIPDDSAFMQEIYTKRNLKLTRIPKDMMHLVPTTLLHSLEGMHGLEWEKLLKLQCSDGSFLFSPSSTAFALMQTKDENCLQYLNKVVQRFNGGVPNVYPVDLFEHIWAVDRLERLGISRYFQSEIKECLNYVYSYWTEDGICWARNSRVHDIDDTAMGFRLLRLHGYDVSADSFRQFEKGGEFFCFAGQSNQAVTGIFNLYRASQVLFPGEKILEDAKTFSFRFLKEKLAANQLLDKWIITKDLPGEVGHAVDIPWYASLPRLESRYYIEQYGGDDDVWIGKTLYRMPLVNNNDYLELAKIDYNHCQALHQLEWISFQKWYTEYNLGEFGVSRETLVQAYFVAAASIFEPERWTERLAWARTGVLVKAVCFYLEKQADQEWRDFVDAFNSSSNNCDNTPKSNTRRWGSKRTAEGLIRALLQTLNRLSLDSLMAHGIHIRHYLQRAWGRWLLTWWEEKQEEEEDRQHEREAELLVLSINLCAGRCISEELLSHPHYARVVQLTNRLFHHLSRFRECKVKEKESNNIPNMRSTTTPLEIESDMQELVQCVLRPSDGIDPAIKQTFLTVAKGYYYTAYCPHDMINRHIAKVLFERIN |
| *NnTPS8* | MGSHLQFTFIKSGGCRSSVLANSFLIPLSSFSTKKFSQGRCLHPVRPLQNAQVIKHIQLHVRSQLSNEEVIRRSGNYKPSSWENDDLQSLKSDFPEEAYVERATKLVVDVKRKFNQAAGAVDLLELIDNVQRLGVGYHFQTEIKRALESIKDTSVNADEELYATALRFRLLRQHGFQISQDVFKSFMDKKGGSFNESLRKDTKGLLDLYEASYHGFEGEDILDGAQQFTKRHLIKQLKEKTSPNYKNMARLVSHALEMPLHWRMNRWEARWFIDTYSMKQDMDPLILEFAKLDFNMVQAIYQQELKDASRWWTDLGLAEALRFSRDRIVENYLWTTGIIFEPQFGYCRRQLTKANCLITTIDDVYDVYGTLDELKLFTDAVERWDINTLRDLPDYMKICFMALYNNINEMAYDFLKKRGWDILKYLKKGWLDLCKTYLVEAQWYYNGYKPTLEEYLNNAWISISAPTILVHNYFCVENKIKEEALEQLDNYPSIVRSSGALLRYSDDLGTSTDELKRGDVPKSIQCYMHETGAPEEIAREHIRGLRAKEWKKMNKCMTAPSPFPQAFKDSAAGLGRMAQFMYQHGDGHGLLADSLTKNRIMPLLVHPIPLD |
| *NnTPS9* | MALSISFSHSSLFSSKKFSQTCDPLARPLQNLHVTSQHVRLFFCSSLVSQFTAAARRSGNYQPSIWDNDFVQSLKTDFTGERYVRWAKKLGEEVRRKFNEVAEPLALLELIDTVERLGMGYHFQTEIKGALETIISIKDNNFCAGEEIYAQALRFRLLRQHGFQVSQDVFKSFKEEVGDGYFTFGGPVCKDIRTILSLYEASYYAFEGESILDEAQRFTRRQLKKKLKENIAPDLTREISHAMELPLHWRMQRMEARWFLDTYGMREDMDPLLLKFAKLDFNLVQATHQEDLKHVSRWWNNLGLGKALSFSRDWLVECFLFTVGAMFQPEFGFGRRQLTKAVSLINVIDDVYDIYGSLDELQLFTEAVQRWDINNLQDLPEYMQICFMALYNTTNELAYDFLKEHGHDILKYLKKEWVDLCKAYLVEAMWHHNGYKPTLEEYMNNAWISVGLPLVLTQNYLCSSRDNITKEALEQLGKYSNIIRSSSLIFRFADDFGTSSDELRRGDVPKSIQCYMHDTDCSEEIAREHIRALVAKTWKQMNEHASAPFPPLFITSAVNFAQTALFMYQYRDGHGVADWKLKDGIMSLLVEPISLMV |
| *NnTPS10* | MSSQISSCPPTQHSSSEAEKTELIRHTTNFHPSIWGDRFITYTCDNMKLESYNQQVEELKEEVRSILRNSANKPLEMLSLIDAVQRLGLGYHFEREINKMLEQIFDAHIVYFNDMDDNLNAVALLFRLLRQHGYNIPCDVFKKFKDEDGKLRESLARDIQGMLSLYEAAYLGKRGEEVLDEALAFTTAHLKSTVATDTTSPTLVKQVKHALEQPLHRGIPKIEARHYISFYEEDKSRNQVLLKLAKLDFNRTQLLYLNELSQVSRWWKNIDFTSKTPYARDRLAECYFWSAGALPEPQYSLGRKTLAKIVAINTILDDTYDAYGTFEELQLLTDAIRRWDANETDKLPEYMKVIYVTLLDIYNEIEEEMRKEGRCFCVSYAKEAMKDLIEAYQIEATWLKEGHIPTFEEFSKLGLITSTYIMMTNVIFACMGEMATKEVFDWLSKTPDAVKSSCIIGRFSDDIMTNEFEQERGHVCSAIECYMKQYGVSREEANNELRKIVTKAWKVLNEECITPTAAPMPLLIPVVNLARLIEVVYKHGDAFTNVTTGLKTHIKLLLIDPITI |
| *NnTPS11* | MLHQASSCPPILCLPEIEKPKVVRRSANFHPSIWGDRFTTYTCDNMTLDACNKQAEGLKEEVRCMLMNSTNGPSEILSLIDTIQRLGLAYHFEREIDEILEHMLVTHVGYFNGVDDDLHTIALWFRLLRQNGYNIPCDIFNKFKDDHGKFRDGLTRDLKGILGLYEAAHLGIRGEEVLDEALAFTTAQLESSVVTNTSSHLVKQVMHALEQPFHRGMLKLEARHYISFYEQDETHNPVLLQFAKLDFNRLQPLHLKELSQISKWWKDLDFALKIPYARNRVVECYFWIMGVYPEPQYSLGRMILTKVIAMTSVLDDTYDIYGTLEELQLFTDAIERWDVGETDQLLEYMKVIYLALLDVYNEIEEQVNREGRSYCVYYAKEAMRKQARAYLVEAKWFFQGHIPIVEEYMDVALISTSYPLLAVASFVGMGEIATKEAFDWVSNLPNLVKNSSIICRLMDDIMTHEYEQEKGDVCSSVECYMKQYGVSKQEVVEEFSRSITNAWKDINEECLRTTAIPKPLLTRVANLARMMDVIYKHGNGYTNAETVLKDHITSLLIDSIKI |

**Table S3. Overview of the RNA-seq information in the NCBI database.**

| **Sample** | **SRA Acc.** | **Sample** | **SRA Acc.** |
| --- | --- | --- | --- |
| Leaf_S1_1 | SRR5118116 | Stamenes_T14_3 | SRR15204541 |
| Leaf_S1_2 | SRR5110884 | Carpels_T2_1 | SRR15204559 |
| Leaf_S1_3 | SRR5115159 | Carpels_T2_2 | SRR15204558 |
| Leaf_S2_1 | SRR5115161 | Carpels_T2_3 | SRR15204557 |
| Leaf_S2_2 | SRR5115163 | Carpels_T14_1 | SRR15204546 |
| Leaf_S2_3 | SRR5115207 | Carpels_T14_2 | SRR15204545 |
| Leaf_S3_1 | SRR5115208 | Carpels_T14_3 | SRR15204544 |
| Leaf_S3_2 | SRR5115209 | Seed_D8_1 | SRR10829966 |
| Leaf_S3_3 | SRR5115210 | Seed_D8_2 | SRR10829969 |
| Leaf_S4_1 | SRR5115211 | Seed_D8_3 | SRR10829970 |
| Leaf_S4_2 | SRR5116526 | Seed_D15_1 | SRR10829963 |
| Leaf_S4_3 | SRR5116527 | Seed_D15_2 | SRR10829964 |
| Petal_S1_1 | SRR13024355 | Seed_D15_3 | SRR10829965 |
| Petal_S1_2 | SRR13024354 | Seed_D20_1 | SRR10829960 |
| Petal_S1_3 | SRR13024343 | Seed_D20_2 | SRR10829961 |
| Petal_S2_1 | SRR13024332 | Seed_D20_3 | SRR10829962 |
| Petal_S2_2 | SRR13024321 | Seed_D25_1 | SRR10829967 |
| Petal_S2_3 | SRR13024312 | Seed_D25_2 | SRR10829968 |
| Petal_S3_1 | SRR13024311 | Seed_D25_3 | SRR10829959 |
| Petal_S3_2 | SRR13024310 | Leaf_1 | SRR5115161 |
| Petal_S3_3 | SRR13024309 | Leaf_2 | SRR5115163 |
| Petal_S4_1 | SRR13024308 | Leaf_3 | SRR5115207 |
| Petal_S4_2 | SRR13024353 | Petal_1 | SRR13024332 |
| Petal_S4_3 | SRR13024352 | Petal_2 | SRR13024321 |
| Stamenes_T2_1 | SRR15204553 | Petal_3 | SRR13024312 |
| Stamenes_T2_2 | SRR15204552 | Seed_1 | SRR8238715 |
| Stamenes_T2_3 | SRR15204550 | Seed_2 | SRR8238716 |
| Stamenes_T14_1 | SRR15204543 | Seed_3 | SRR8238717 |
| Stamenes_T14_2 | SRR15204542 |  |  |

**Table S4. Prenyltransferase (PT) genes used in this study.**

| **Prenyltransferase** | **Gene Name** | **GenBank Acc.** | **Reference** |
| --- | --- | --- | --- |
| GPPS | *ERG20_F96W-N127W_* | NP_012368 | 1,2 |
| NPPS | *NDPS1* | QNM36897 | 2 |
| E,E-FPPS | *ERG20* | NP_012368 | 3 |
| Z,Z-FPPS | *SlCPT6* | K7W9N9 | 4 |
| GGPPS | *ERG20_F96C_* | NP_012368 | 5 |

**Reference**

1. Ignea, C., Pontini, M., Maffei, M. E., Makris, A. M. & Kampranis, S. C. Engineering monoterpene production in yeast using a synthetic dominant negative geranyl diphosphate synthase. *Acs Synth. Biol*. **3**, 298 (2014).

2. Zhao, J., Bao, X., Li, C., Shen, Y. & Hou, J. Improving monoterpene geraniol production through geranyl diphosphate synthesis regulation in *Saccharomyces cerevisiae*. *Appl Microbiol Biotechnol*. **100**, 4561 (2016).

3. Lu, C., Zhang, C., Zhao, F., Li, D. & Lu, W. Biosynthesis of ursolic acid and oleanolic acid in *Saccharomyces cerevisiae*. *Aiche J*. **64**, 3794 (2018).

4. Akhtar, T. A. et al. The tomato cis-prenyltransferase gene family. *Plant J*. **73**, 640 (2013).

5. Ignea, C. et al. Efficient diterpene production in yeast by engineering Erg20p into a geranylgeranyl diphosphate synthase. *Metab Eng*. **27**, 65 (2015).

**Table S5. Primers used in this study.**

| **Name** | **Primer (5'-3')** |
| --- | --- |
| β-Actin-qPCR-F | TCGGTTGGACCTTGCT |
| β-Actin-qPCR-R | CCATCAGGCAGCTCGTA |
| NnTPS5-qPCR-F | GTCGGAGACACGCTTTCAGA |
| NnTPS5-qPCR-R | CATTCAGGAGACGGCCACAT |
| NnTPS6-qPCR-F | GCGGGAAACAGAGGAAGGAA |
| NnTPS6-qPCR-R | CCAGCAGTGAGTCCAACGAT |
| NnTPS7-qPCR-F | AGAGCAATATGGTGGGGACG |
| NnTPS7-qPCR-R | TGCTAGCTCAAGATAGTCATTGTT |
| NnTPS1-pZY-F | CGTCGTCATCCTTGTAATCAAGTTTGTCAAGTGGAATTG |
| NnTPS1-pZY-R | TTTGAAAATTCAATATAAATGTCAGCCAACTCCTTTTTT |
| NnTPS2-pZY-F | CGTCGTCATCCTTGTAATCAGAGACAGACAAAAAAGAAC |
| NnTPS2-pZY-R | TTTGAAAATTCAATATAAATGGCATTGAAATTGAAC |
| NnTPS3-pZY-F | CGTCGTCATCCTTGTAATCACCTCTCAATGGAATATTTTC |
| NnTPS3-pZY-R | TTTGAAAATTCAATATAAATGTCATTCTTGTCCATTATC |
| NnTPS4-pZY-F | CGTCGTCATCCTTGTAATCAATGGTAATAGGGTCGATTAG |
| NnTPS4-pZY-R | TTTGAAAATTCAATATAAATGTCATCCCAAATTTCCTC |
| NnTPS5-pZY-F | CGTCATCCTTGTAATCCATAGAAGACAACAAACCAGTAC |
| NnTPS5-pZY-R | TTTGAAAATTCAATATAAATGTCATTGATTTACCCAAAC |
| NnTPS6-pZY-F | CGTCGTCATCCTTGTAATCATGAACAGATTCAAACAAAAC |
| NnTPS6-pZY-R | TTTGAAAATTCAATATAAATGCCAAACACAACAGCATTC |
| NnTPS7-pZY-F | CGTCGTCATCCTTGTAATCGTTTATTCTTTCAAACAGC |
| NnTPS7-pZY-R | TTTGAAAATTCAATATAAATGATTCGTACCATGAACG |
| NnTPS8-pZY-F | CGTCGTCATCCTTGTAATCATCCAGTGGGATTGGGT |
| NnTPS8-pZY-R | TTTGAAAATTCAATATAAATGGGTTCTCATCTGCAGT |
| NnTPS9-pZY-F | CGTCGTCATCCTTGTAATCGACCATCAAAGAAATTGGTT |
| NnTPS9-pZY-R | TTTGAAAATTCAATATAAATGGCATTGAGCATCAGCT |
| NnTPS10-pZY-F | CGTCGTCATCCTTGTAATCGATCGTAATCGGATCAATC |
| NnTPS10-pZY-R | TTTGAAAATTCAATATAAATGAGCTCCCAAATTTCTAG |
| NnTPS11-pZY-F | CGTCGTCATCCTTGTAATCGATTTTAATGCTATCGATC |
| NnTPS11-pZY-R | TTTGAAAATTCAATATAAATGTTGCATCAGGCTTCCTC |
| NnTPS6-II-F | CAATCTAATCTAAGTTTTCTAGATGCCAAACACAACAGC |
| NnTPS6-II-R | GTAATCTGGAACATCGTATGGGTAATGAACAGATTCAAAC |
| NnTPS7-II-F | CAATCTAATCTAAGTTTTCTAGATGATTCGTACCATGAACG |
| NnTPS7-II-R | GTAATCTGGAACATCGTATGGGTAGTTTATTCTTTCAAACAG |
| Act 1-F | GACCATGATTACGAATTCGTAGCTAGCATACTCGAGGTC |
| Act 1-R | GGTACCGAGCTCGAATTCCTTCTACCTACAAAAAAGCTCC |
| Act 2-F | GACCATGATTACGAATTCGACCAGGACAAAATTTAGAACGA |
| Act 2-R | GGTACCGAGCTCGAATTCTTTTTATGAGCTGCAAACACAC |
| AtHMGR-F | AATTCGAGCTCGGTACGTCGACATGGTCACGTGCTCTC |
| AtHMGR-R | GTATCCCATCTCGGTACGTCGACGTGTTGTTGTTGTTGTCGT |
| AtFPPS-F | GGATCCTCTAGAGTCGATCATGGCGGATCTGAAATCA |
| AtFPPS-R | ATCTCGGTACCGTCGAGCTTCTGCCTCTTGTAGA |
| Act 2-AtFPS-F | GTTTCCCGCCTTCAGTTTCCAGGACAAAATTTAGAACGAAC |
| Act 2-AtFPS-R | CCTGTCAAACACTGATAGTTTCCAGTGCCACTTATCTTTAA |
| AtDXS-F | TGAAATCTAGAGTCGAAATGGCTTCTTCTGCATTTGC |
| AtDXS-R | ATCTCGGTACCGTCGACAAACAGAGCTTCCCTTG |
| tp-F | TACCCGGGGATCCTCTAGTTTAATGGCTTCCTCTATGC |
| tp-R | GTACCGTCGACTCTAGATTTCAAACCGGTGAAGCTTGGTG |
| AtGPPS-F | ACCGGTGTGAAAGGTACAATGTTATTCACGAGGAGTG |
| AtGPPS-R | TATCCCATCTCGGTACCCTTGTTTCTGGTGATGAC |
| AtGGPPS-F | ACCGGTGTGAAAGGTACAATGGCTTCAGTGACT |
| AtGGPPS-R | TATCCCATCTCGGTACCGTTCTGTCTATAGGC |
| NnTPS3-pEAQ-F | TATTCTGCCCAAATTCGTCGACATGTCATTCTTGTCCATTAT |
| NnTPS3-pEAQ-F | TCGGTACCAAGCTTGTCGACACCTCTCAATGGAATATTTTC |
| NnTPS10-pEAQ-F | TATTCTGCCCAAATTCGTCGACATGAGCTCCCAAATTTCTA |
| NnTPS10-pEAQ-F | TCGGTACCAAGCTTGTCGACGATCGTAATCGGATCAATC |
| NnTPS6-pEAQ-F | TATTCTGCCCAAATTCGTCGACATGCCAAACACAACAGC |
| NnTPS6-pEAQ-F | TCGGTACCAAGCTTGTCGACATGAACAGATTCAAAC |
| NnTPS10(1-164)-F | CAAGGTATGTTGTCCTTGTACG |
| NnTPS10(1-164)-R | GACAACATACCTTGGATGTCAC |
| NnTPS10(165-564)-F | CAAGGTATGTTGTCTTTATACG |
| NnTPS10(165-564)-R | GACAACATACCTTGAATATCTC |
| NnTPS10-A530T-F | GTTAATCTGACTAGATTGATCGAAGTTG |
| NnTPS10-A530T-R | CAATCTAGTCAGATTAACGACAGGAA |
| NnTPS10-N313T-F | CCATCACAACCATTCTGGATG |
| NnTPS10-N313T-R | AATGGTTGTGATGGCAACGATTT |
| NnTPS10-V310I-F | AATCATCGCCATCAATACCATTC |
| NnTPS10-V310I-R | GATGGCGATGATTTTAGCCAGGGT |
| NnTPS10-V536I-F | AAGTTATTTATAAACATGGTGATG |
| NnTPS10-V536I-R | GTTTATAAATAACTTCGATCAATC |
| NnTPS4-F530L-F | GGTTAATCTGACTAGATTGGCAGAC |
| NnTPS4-F530L-R | AATCTAGTCAGATTAACCAAGGGCA |
| NnTPS4-I311V-F | AAAGATCGTTGCCATTACAACCAT |
| NnTPS4-I311V-R | AATGGCAACGATCTTTGCCAAAAT |
| NnTPS4-T314N-F | GCCATTAATACCATTTTGGATGATA |
| NnTPS4-T314N-R | AATGGTATTAATGGCGATGATCTT |
| NnTPS4-V294A-F | GCAGGTGCTTATCCTGAACCACAATAC |
| NnTPS4-V294A-R | AGGATAAGCACCTGCTGACCAAAAATAAC |
| NnTPS4-Y295L-F | GCAGGTGTTTTGCCTGAACCACAATAC |
| NnTPS4-Y295L-R | AGGCAAAACACCTGCTGACCAAAAATAAC |
| NnTPS10- pET28a-F | ACTTTAAGAAGGAGATATACATGAGCTCCCAAATTTCTAG |
| NnTPS10- pET28a-F | AGCTTGTCGACGGAGCTCGAGATCGTAATCGGATCAATC |

**Table S6. Plasmids and strains used in this study.**

| **Name** | **Description** | **Type** | **Source** |
| --- | --- | --- | --- |
| p912 | pRS426,  *leu2Δ::LEU2_*P*_GAL1__ERG20(F96W,N127W)_*T*_ERG20_*, P*_GAL10__LacZ-FLAG_*T*_CYC1_*, *URA3(MssI)* | Plasmid | 1 |
| p916 | pRS426,  *leu2Δ::LEU2_*P*_GAL1__tNPPs-HA_*T*_ERG20_*, P*_GAL10__LacZ-FLAG_*T*_CYC1_*, *URA3(MssI)* | Plasmid | 1 |
| p900 | pRS426, *leu2Δ::LEU2_*P*_GAL1__ERG20_*T*_ERG20_*, P*_GAL10__LacZ-FLAG_*T*_CYC1_*, *URA3(MssI)* | Plasmid | 1 |
| p923 | pRS426,  *leu2Δ::LEU2_*P*_GAL1__ZFPPs-HA_*T*_ERG20_*, P*_GAL7__tNPPs_*T*_DH3,_*  P*_GAL10__LacZ-FLAG_*T*_CYC1_*, *URA3(MssI)* | Plasmid | 1 |
| p914 | pRS426, *leu2Δ::LEU2_*P*_GAL1__ERG20(F96C)_*T*_ERG20_*, P*_GAL10__LacZ-FLAG_*T*_CYC1_*, *URA3(MssI)* | Plasmid | This study |
| pYJ900 | pRS426, *leu2Δ::LEU2_*P*_GAL1__ERG20_*T*_ERG20_*, P*_GAL10__NnTPS10-FLAG_*T*_CYC1_ (MssI)* | Plasmid | This study |
| pYJ905 | pRS426, *URA3Δ::HIS3_*P*_GAL1__NnTPS10-FLAG_*T*_PGK1_*, P*_GAL10__tHMG1_*T*_CYC1_ (MssI)* | Plasmid | This study |
| pYJ908 | pRS426, *YPRCdelta15Δ::TRP_*P*_GAL1__NnTPS10-FLAG_*T*_PGK1_*, P*_GAL10__*T*_GPM1_ (NotI)* | Plasmid | This study |
| pCas | 2μm, ampR, *TEF1p-iCas9, SNR52p* | Plasmid | 2 |
| pHM012 | pCas9, derivative with gRNA targeting at the X-4,  XI-1 and XII-5 chromosome | Plasmid | 1 |
| pEAQ-HT | pBINPLUS, *KanR::*P*_CaMV 35S__P19_*T*_CaMV_* *_poly(A) signal_* | Plasmid | 3 |
| JCR27 | CEN.PK2-1D  *ChrXII-2Δ::HphMX_*P*_TEF1__cas9_*T*_CYC1_*,  *ChrXI-3Δ::*P*_GAL1__ERG8_*T*_CYC1_*, P*_GAL10__tHMG1_*T*_GAL10_*, P*_GAL7__ERG12_*T*_ADH1_*,  *ChrX-3Δ::*P*_GAL1__ERG13_*T*_CYC1_*,P*_GAL10__tHMG1_*T*_HMG1_*, *ChrXII-4Δ::*P*_GAL1__IDI1_*T*_CYC1_*, P*_GAL10__ERG10_*T*_GAL10_*, P*_GAL7__MVD1_*T*_ADH1_* | Strain | 4 |
| BL21(DE3)pLysS | 1. *omp*T *hsd*S_B_(r_B_^-^, m_B_^-^) *gal dcm*(DE3)pLysS Cam^r^ | Strain |  |
| GV3101 | C58 (rifR) Ti pMP90 (pTiC58DT-DNA) (GentR) Nopaline | Strain |  |
| JE1 | JCR27  *leu2Δ::*P*_GAL1__ERG20_*T*_ERG20_*, P*_GAL10__NnTPS10-FLAG_*T*_CYC1_*,*LEU*,  *YPRCdelta15Δ::*P*_GAL1__NnTPS10-FLAG _*T*_PGK1_*, *TRP*,  *URA3Δ::*P*_GAL1__NnTPS10-FLAG_*T*_PGK1_*,P*_GAL10__tHMG1_*T*_CY\C1_, HIS* | Strain | This study |
| JE2 | JCR27  *leu2Δ::*P*_GAL1__ERG20_*T*_ERG20_*, P*_GAL10__NnTPS10-FLAG_*T*_CYC1_*,*LEU*,  *YPRCdelta15Δ::*P*_GAL1__NnTPS10-FLAG _*T*_PGK1_*, *TRP*,  *URA3Δ::*P*_GAL1__NnTPS10-FLAG_*T*_PGK1_*,P*_GAL10__tHMG1_*T*_CYC1_, HIS*  *gal80Δ::URA3* | Strain | This study |

**Reference**

1. Zhi, Y. et al. Gene-directed in vitro mining uncovers the insect-repellent constituent from mugwort (*Artemisia argyi*). *J. Am. Chem. Soc*. **146**, 30883 (2024).

2. Zhang, Y. et al. A gRNA-tRNA array for CRISPR-Cas9 based rapid multiplexed genome editing in *Saccharomyces cerevisiae*. *Nat. Commun*. **10**, 1010 (2019).

3. Sainsbury, F., Thuenemann, E. C. & Lomonossoff, G. P. pEAQ: versatile expression vectors for easy and quick transient expression of heterologous proteins in plants. *Plant Biotechnol J*. **7**, 682 (2009).

4. Siemon, T. et al. Semisynthesis of plant-derived englerin A enabled by microbe engineering of guaia-6,10(14)-diene as building block. *J. Am. Chem. Soc*. **142**, 2760 (2020).

**Table S7. Cas9 target sites used in this study.**

| ***S. cerevisiae* locus** | **Cas9 target site** |
| --- | --- |
| Cas9 Target Site 1 (X-4) | TCAAGAGCAGCAACAGGCATGGG |
| Cas9 Target Site 2 (XI-1) | GCAATGCGATGTTAGTTTAGTGG |
| Cas9 Target Site 3 (XII-5) | TTGTCACAGTGTCACATCAGCGG |

**Table S8. Abbreviations used in this article.**

| **Abbreviation** | **Full name** |
| --- | --- |
| TPS | terpene synthase |
| MVA | mevalonate |
| MEP | methylerythritol-4-phosphate |
| DMAPP | dimethylallyl diphosphate |
| IPP | isopentenyl diphosphate |
| PT | prenyltransferase |
| GPPS | geranyl pyrophosphate synthase |
| FPPS | farnesyl pyrophosphate synthase |
| GGPPS | geranylgeranyl pyrophosphate synthase |
| GPP | geranyl pyrophosphate |
| FPP | farnesyl pyrophosphate |
| GGPP | geranylgeranyl pyrophosphate |
| NPP | nerolidyl diphosphate |
| NPPS | nerolidyl diphosphate synthase |
| CPP | copalyl diphosphate |
| GA | gibberellin |
| NMR | nuclear magnetic resonance |
| GC-MS | gas chromatography-mass spectrometry |
| CRISPR | clustered regularly interspaced short palindromic repeats |
| PEG | polyethylene glycol |
| TLC | thin-layer chromatography |
| DMSO | dimethyl sulfoxide |
| PDA | potato dextrose agar |
| PCR | polymerase chain reaction |
| YBN | Yeast nitrogen base |
| YPD | Yeast extract peptone dextrose |

**Supplementary Figures**


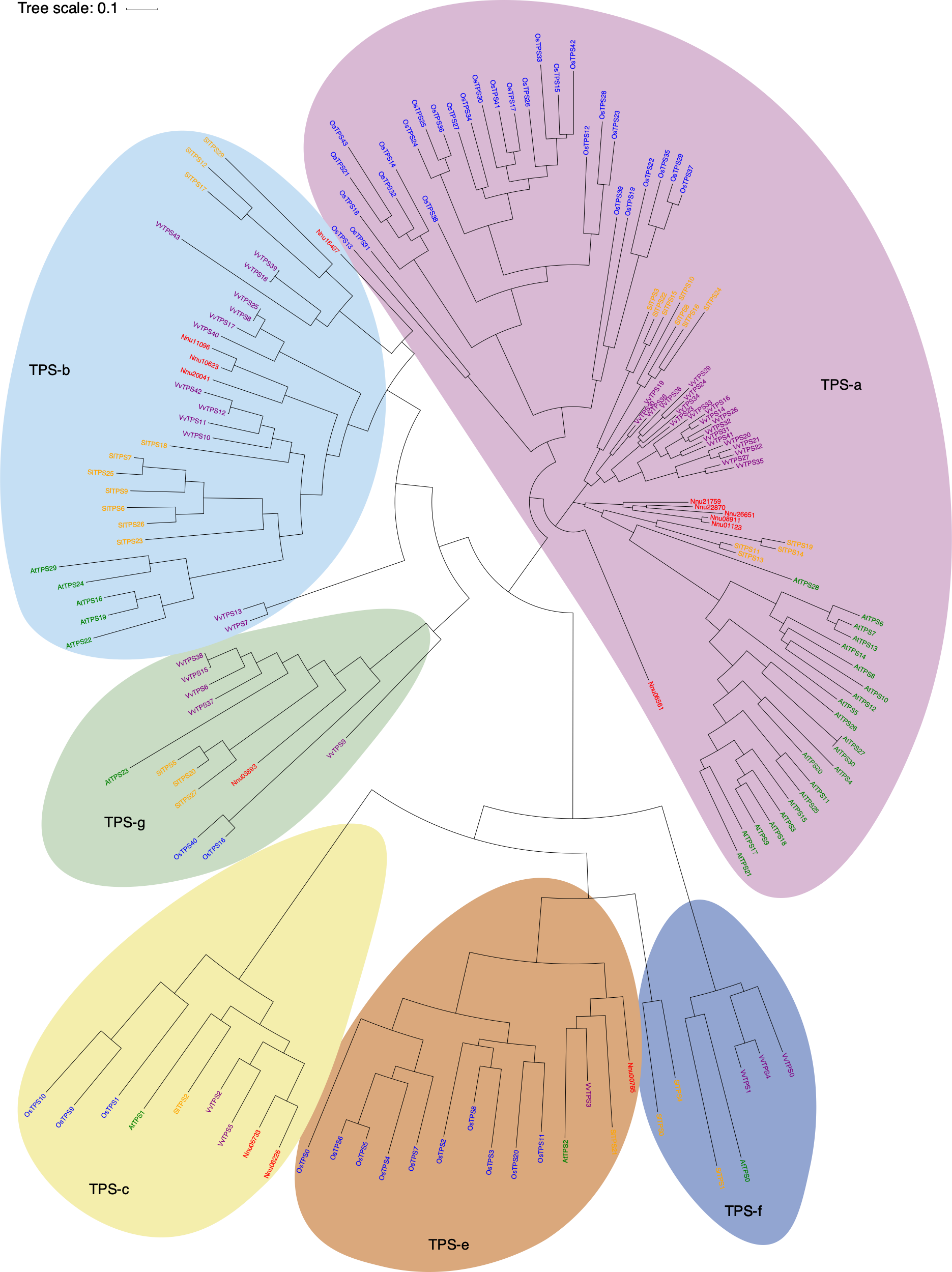


**Figure S1. Phylogenetic analysis of 162 candidate TPSs from five plants.** The tree illustrates the categorization of TPSs into six distinct families, TPS-a, TPS-b, TPS-c, TPS-e, TPS-f, and TPS-g. The TPS distribution across species is as follows: *Nelumbo nucifera* (14 TPSs, red); *Arabidopsis thaliana* (31 TPSs, green); *Oryza sativa* (44 TPSs, blue); *Vitis vinifera* (44 TPSs, purple); and *Solanum lycopersicum* (29 TPSs, yellow).


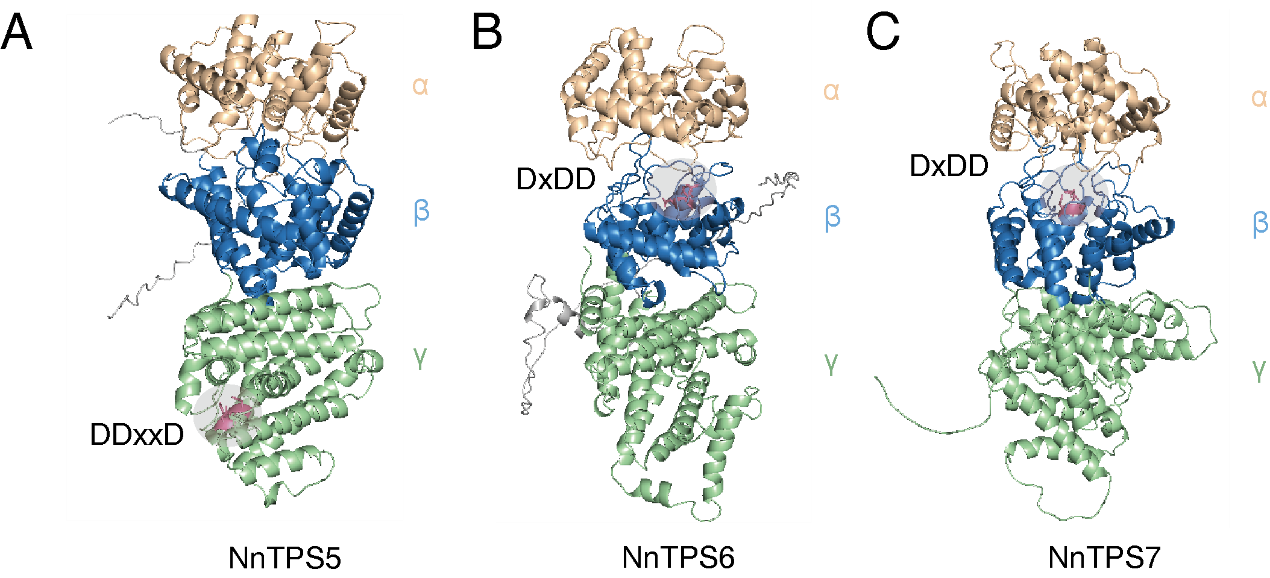


**Figure S2. Predicted Tertiary Structure of NnTPS5 (A), NnTPS6 (B) and NnTPS7 (C).** Conserved DDXXD and DXDD motifs are indicated. The α domain is noted in yellow, while the β domain is in blue, and the γ domain is in green.**
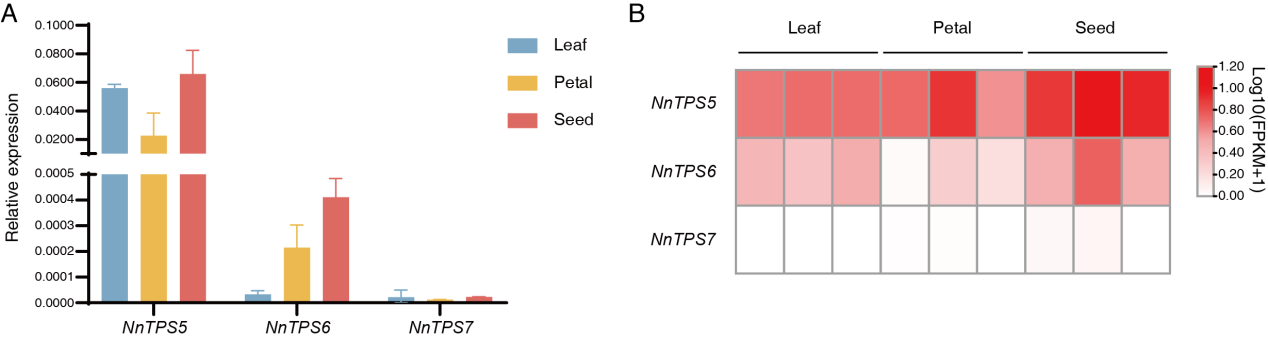
**

**Figure S3. Quantitative real-time PCR of *NnTPS5*, *NnTPS6* and *NnTPS7*.** (A) Quantitative real-time PCR (qRT-PCR) analysis of relative expression levels of *NnTPS5*, *NnTPS6* and *NnTPS7* in sacred lotus (*Nelumbo nucifera*) tissues: folded leaves, petals (one day before pollination), and developing seeds (ten days after pollination). *NnActin* (GeneID: XM-010243420.1) was used as the internal reference gene. Data are represented as mean ± SD of three biological replicates. (B) Heatmap representation of FPKM (Fragments Per Kilobase Million) expression values for *NnTPS5*, *NnTPS6* and *NnTPS7* across examined tissues. Color gradient indicates log10(FPKM+1). Complete transcriptome data are available in Table S3.

**
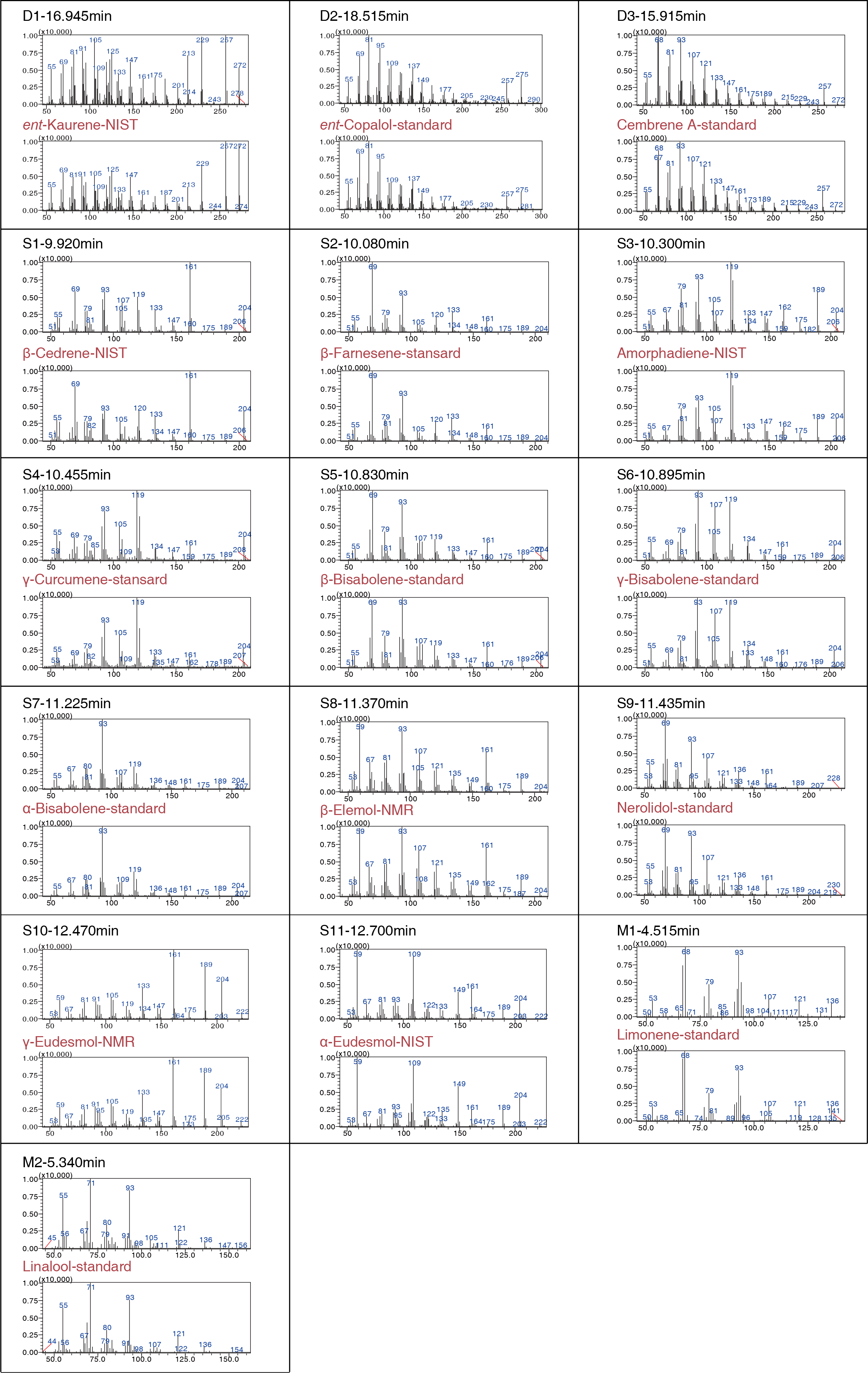
**

**Figure S4. Mass spectrum comparison of NnTPS products and their corresponding authorized compounds.**


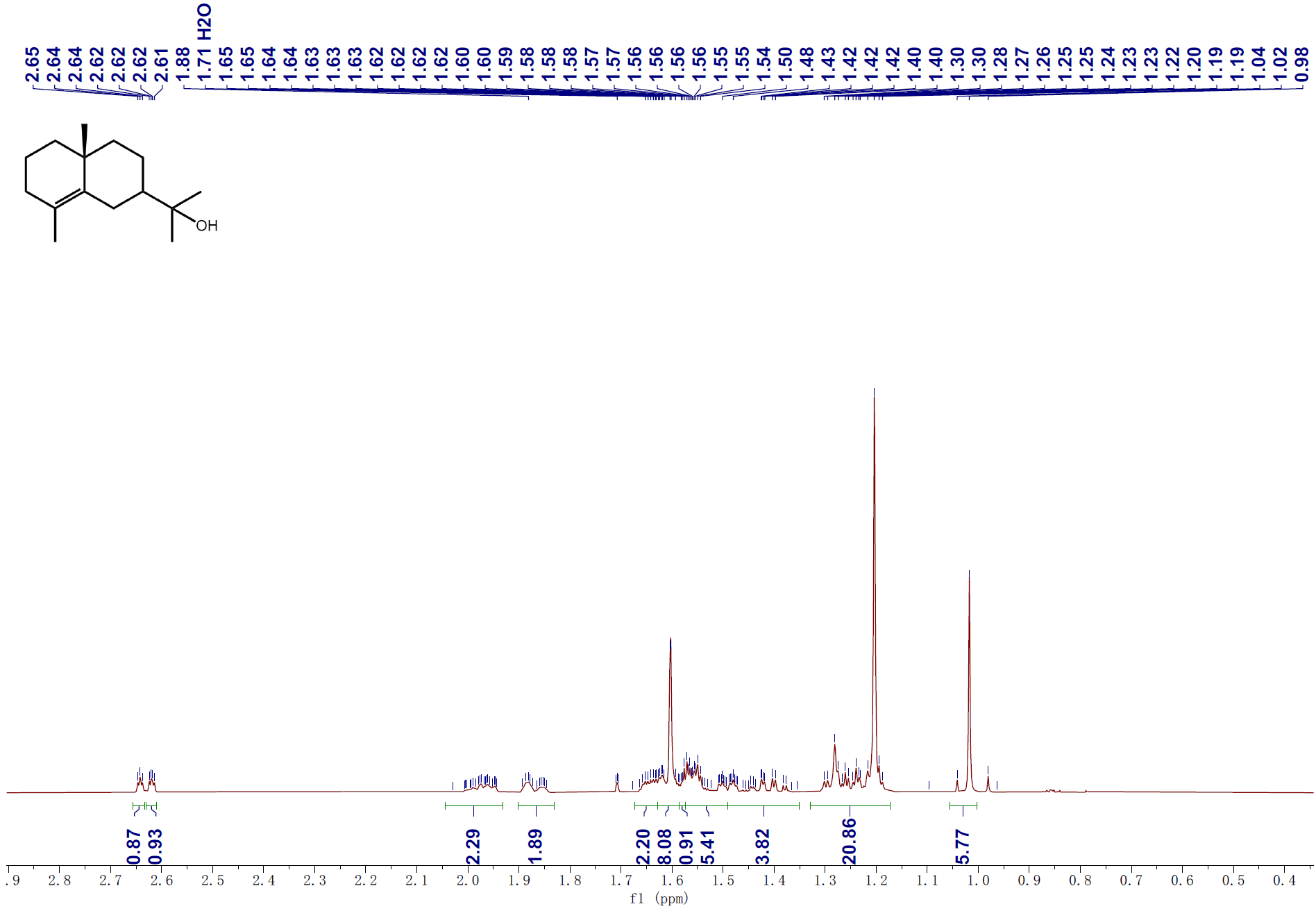


**Figure S5. ^1^H NMR spectrum of γ-eudesmol (CDCl_3_, 600 MHz).** ^1^H NMR (600 MHz, CDCl_3_) *δ* 2.64 (t, *J* = 2.7 Hz, 1H), 2.63 – 2.61 (m, 1H), 2.04 – 1.93 (m, 2H), 1.90 – 1.83 (m, 2H), 1.64 (dt, *J* = 9.8, 3.4 Hz, 2H), 1.63 – 1.59 (m, 8H), 1.58 (d, *J* = 3.3 Hz, 1H), 1.57 – 1.49 (m, 5H), 1.49 – 1.35 (m, 4H), 1.33 – 1.17 (m, 21H), 1.02 (s, 6H).


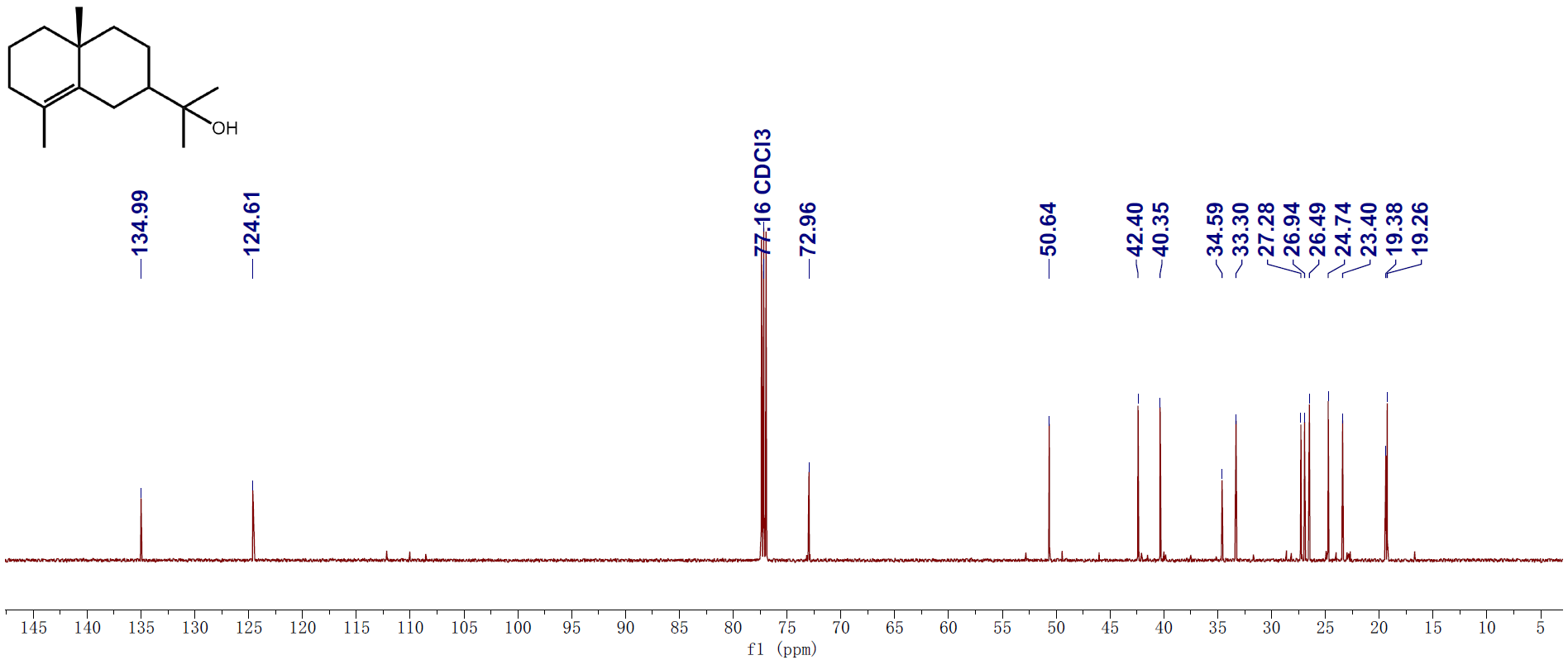


**Figure S6. ^13^C NMR spectrum of γ-eudesmol (CDCl_3_, 151 MHz).** ^13^C NMR (151 MHz, CDCl_3_) *δ* 134.99, 124.61, 72.96, 50.64, 42.40, 40.35, 34.59, 33.30, 27.28, 26.94, 26.49, 24.74, 23.40, 19.38, 19.26.

**
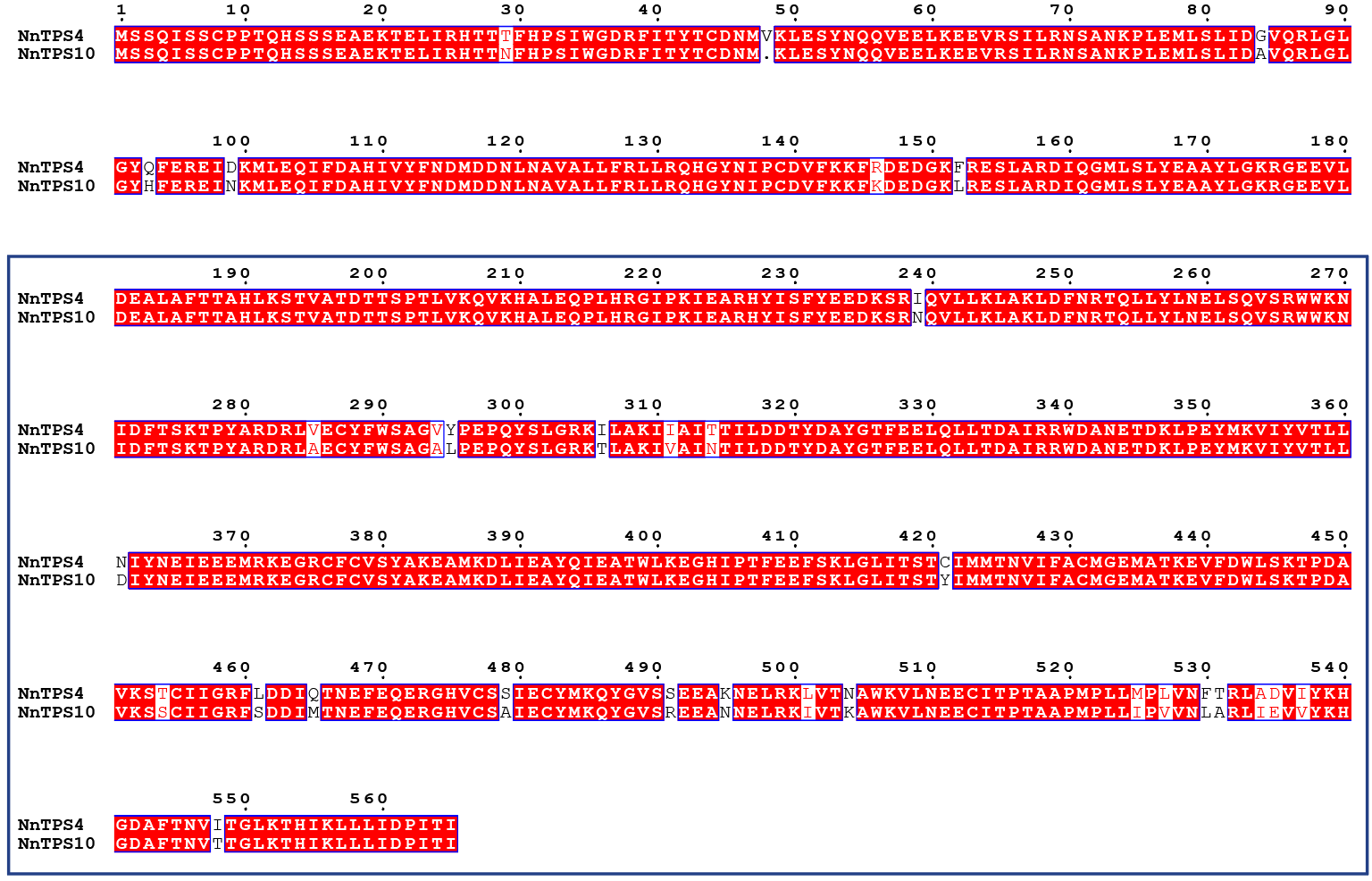
**

**Figure S7. Multiple sequence alignment of NnTPS4 and NnTPS10 by CLUSTALW.** Sequence-conserved regions are marked by shading.


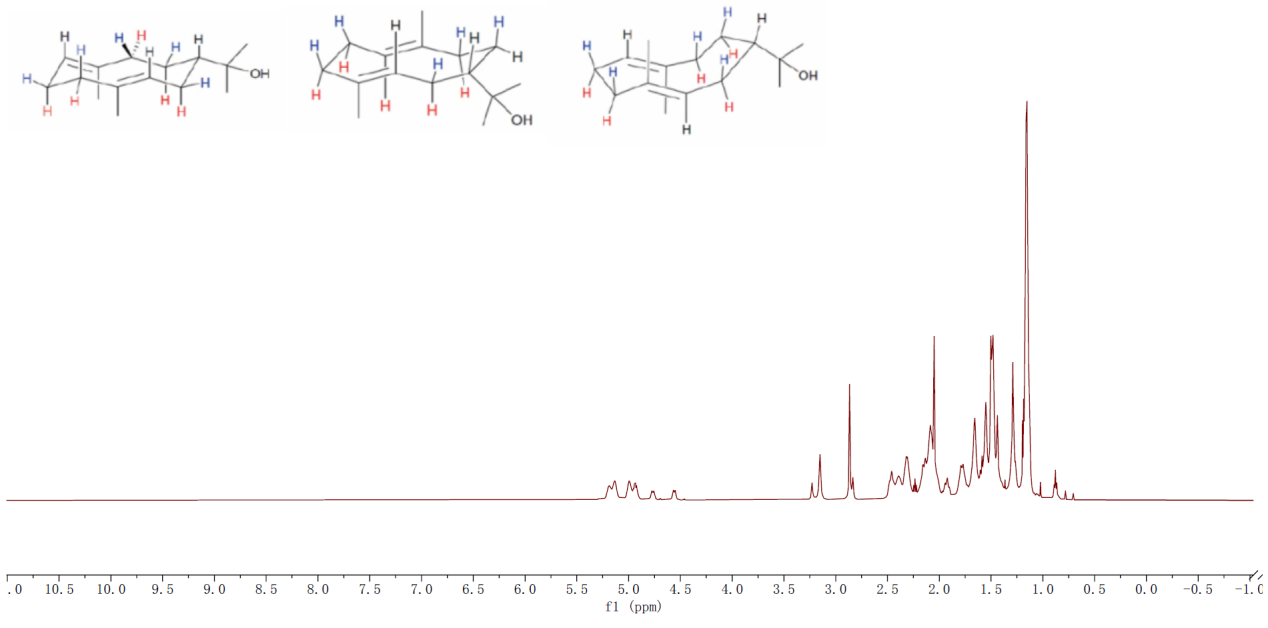


**Figure S8. ^1^H NMR spectrum of hedycaryol ((CD_3_)_2_CO, 600 MHz).** ^1^H NMR (600 MHz, (CD_3_)_2_CO) *δ* 5.27 – 4.88 (m, 1H), 3.37 – 2.77 (m, 1H), 2.54 – 2.27 (m, 2H), 2.20 – 1.88 (m, 3H), 1.84 – 1.35 (m, 6H), 1.35 – 1.08 (m, 6H).


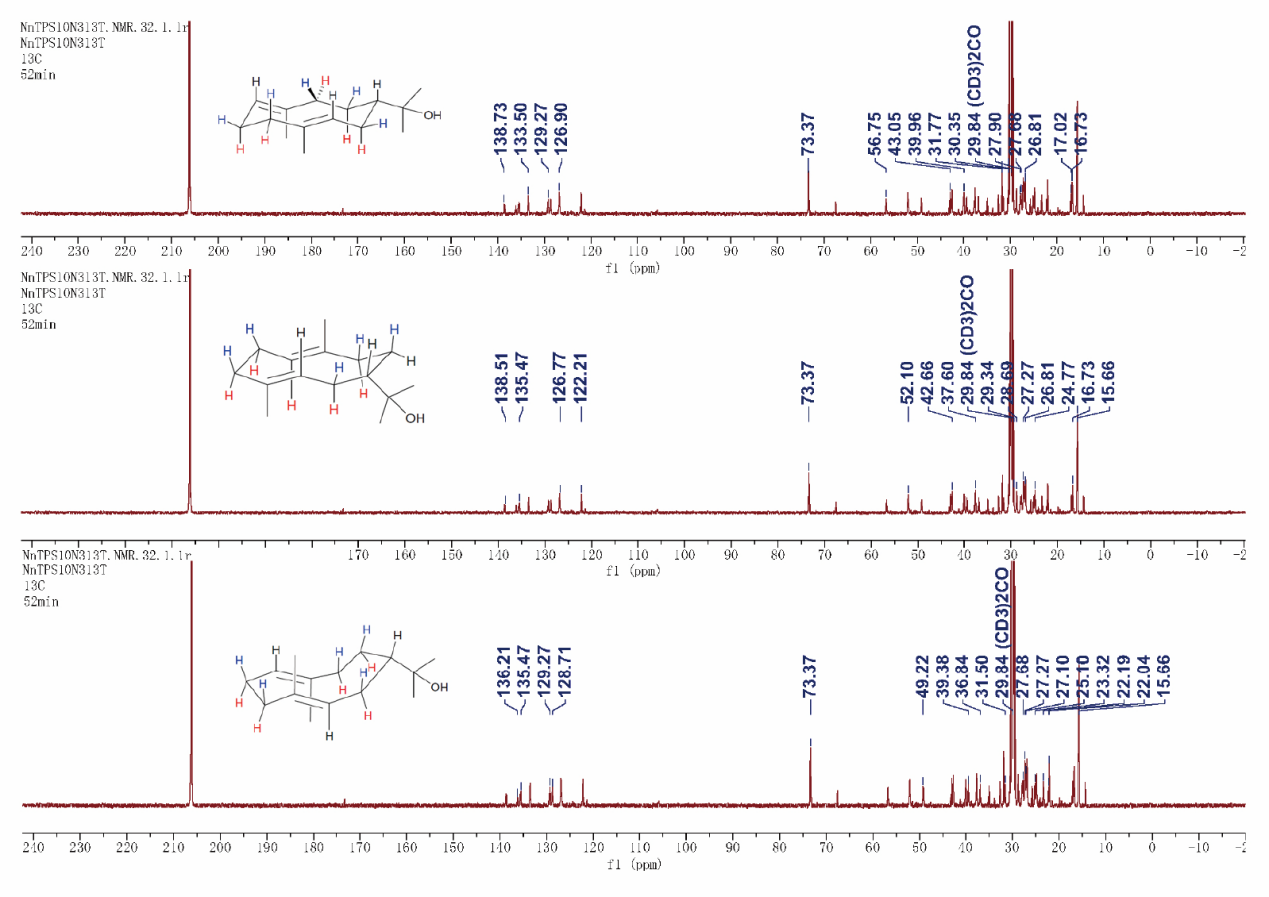


**Figure S9. ^13^C NMR spectrum of hedycaryol ((CD_3_)_2_CO, 151 MHz).** ^13^C NMR (151 MHz, (CD_3_)_2_CO) *δ* 138.73, 133.50, 129.27, 126.90, 73.37, 56.75, 43.05, 39.96, 31.77, 30.35, 27.90, 27.68, 26.81, 17.02, 16.73; ^13^C NMR (151 MHz, (CD_3_)_2_CO) *δ* 138.51, 135.47, 126.77, 122.21, 73.37, 52.10, 42.66, 37.60, 29.34, 28.69, 27.27, 26.81, 24.77, 16.73, 15.66; ^13^C NMR (151 MHz, (CD_3_)_2_CO) *δ* 136.21, 135.47, 129.27, 128.71, 73.37, 49.22, 39.38, 36.84, 31.50, 27.68, 27.27, 27.10, 25.10, 23.32, 22.19, 22.04, 15.66.


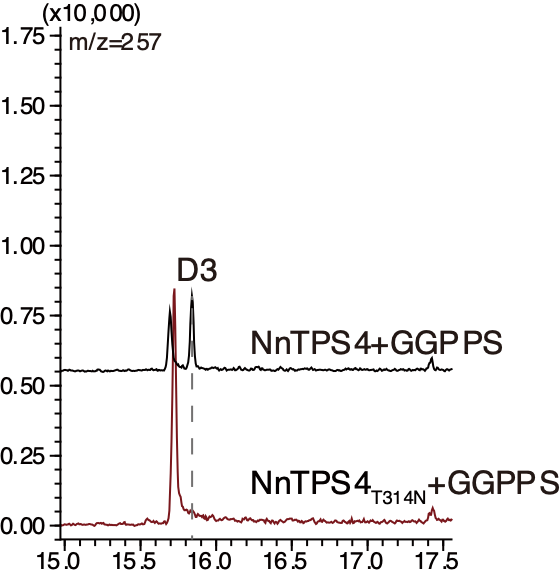


**Figure S10. Gas-chromatography (GC) spectrum profile of products from NnTPS4 and its mutant co-expressed with GGPPS.**

**
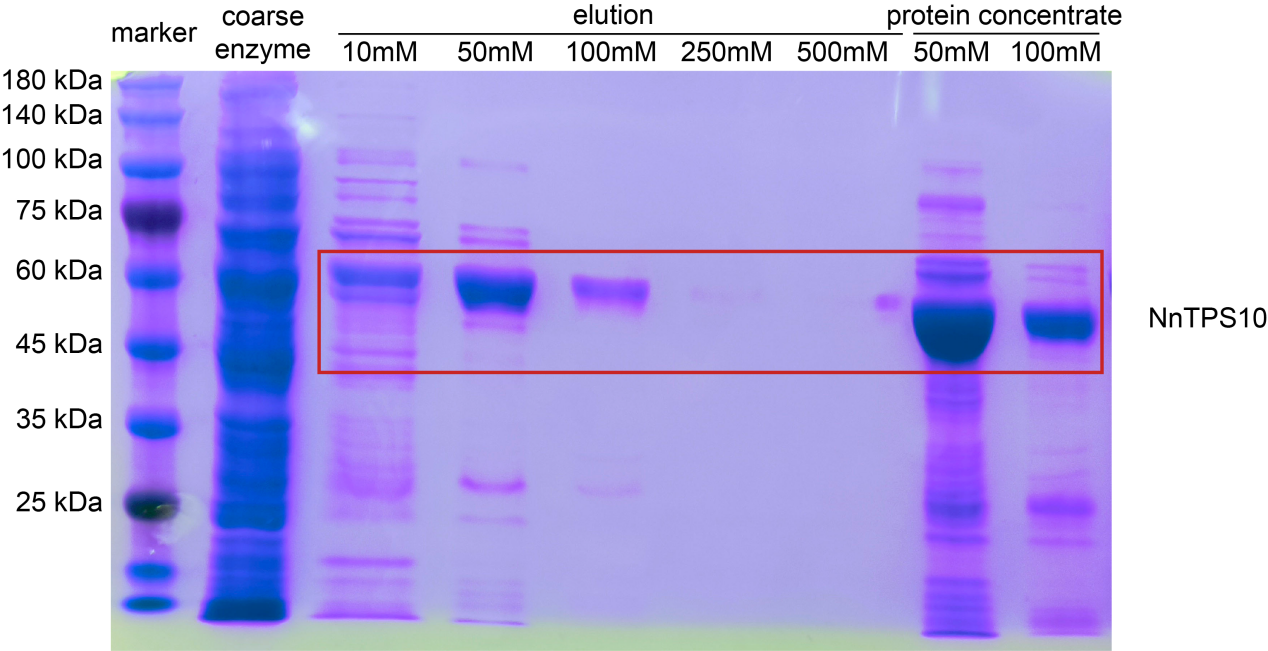
**

**Figure S11. SDS-PAGE of the recombinant NnTPS10 enzymes expressed in *Escherichia coli*.** Proteins were separated on 10% SDS-PAGE and stained with coomassie brilliant blue G-250.


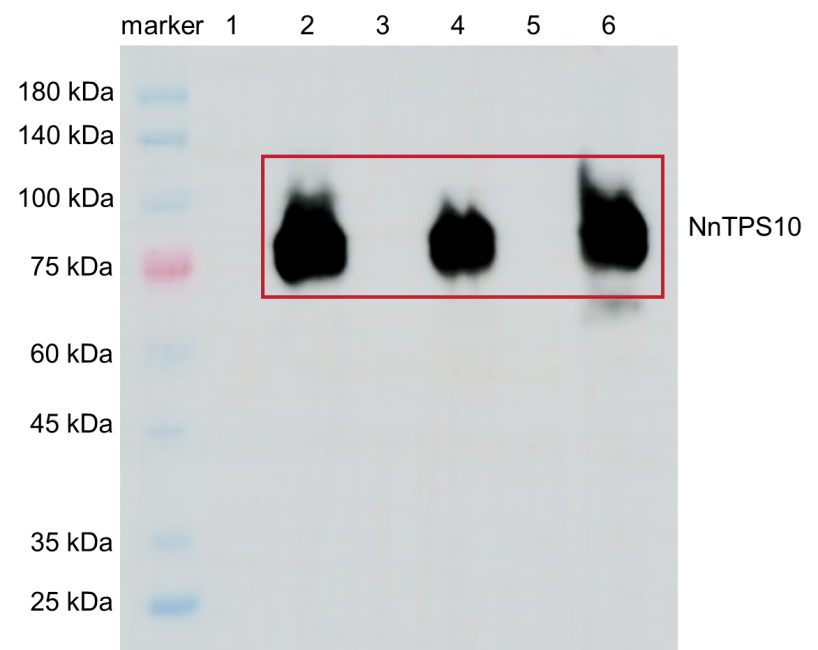


**Figure S12. Western Blot of NnTPS10 expression in *Nicotiana benthamiana*.** Total protein extracts from *N. benthamiana* leaves were separated by 10% SDS-PAGE and transferred to a PVDF membrane. The membrane was probed with a mouse monoclonal anti-Flag primary antibody (ABclonal, Cat. NO: AE005, 1:5,000 dilution) followed by HRP-conjugated goat anti-mouse IgG(H+L) secondary antibody (ABc lonal, Cat. NO: AS003, 1:5,000 dilution). Detection was performed using enhanced chemiluminescence (ECL). Lane1, CK-1; Lane 2, NnTPS10-1; Lane 3, CK-2; Lane 4, NnTPS10-2; Lane 5, CK-3; Lane 6, NnTPS10-3.
